# Supplementary material for: Intravenous immunoglobulin treatment in childhood encephalitis (IgNiTE): a randomised controlled trial
Source: BMJ Open. 2023 Nov 9;13(11):e072134. doi: 10.1136/bmjopen-2023-072134 (PMC10649701; doi:10.1136/bmjopen-2023-072134)
Supplement: Supplementary data [file bmjopen-2023-072134supp001.pdf]

Supplementary Material

Secondary outcomes

Clinical

Secondary clinical outcomes were obtained from routinely collected medical information. These comprised admission to intensive care unit, invasive ventilation requirement, and length of hospital stay, defined as the number of days from admission to a recruiting hospital to discharge from acute care (i.e. not including days in hospital for neurorehabilitation). At 6 and 12 months after randomisation, information on new diagnosis of epilepsy and need for anti-epileptic treatment since discharge were collected.

Neurological and Functional

Secondary neurological outcomes were assessed using age appropriate questionnaires and outcome scores which comprised the GOS-E Peds (assessed at 6 months after randomisation), Liverpool Outcome Score (LOS), Pediatric Quality of Life Score (PedsQL), Gross Motor Function and Classification System (GMFCS), Strengths and Difficulty Questionnaire (SDQ), and Adaptive Behaviour Assessment System, second edition (ABAS-II), all assessed at 4-8 weeks after discharge from acute care and 12 months after randomisation.

The LOS is a validated tool for assessing level of disability after encephalitis in infants and children. It was originally designed to assess disease burden following JE and its use has been extended to other forms of encephalitis. For each participant, a total score (sum of scores for all questions) and an outcome score (the lowest score for any single question) were documented. Based on the outcome score only, participants were assigned to one of 5 outcome categories: 5-Full recovery, 4-Minor sequelae, 3-Moderate sequelae, 2-Severe sequelae, and 1-Death. ‘Good recovery’ was defined as a LOS of 5 and a score of ≤ 4 indicated ‘poor recovery’.

The PedsQL is a brief measure of health-related quality of life comprised of 23 items assessing quality of life in 4 domains: physical functioning (8 items), emotional functioning (5 items), social functioning (5 items) and school functioning (5 items). Based on the scores in each domain, two summary scores (physical health and

psychosocial health summary scores) as well as a total scale score were computed. Total scale scores are presented. A higher total scale score indicates better quality of life.

The GMFCS is an assessment tool based on self-initiated movement and assesses motor function in three areas - walking, sitting, and standing. It uses 5 levels to describe the motor function limitations, taking into consideration age, the use of mobility aids and the quality of movement and is rated from Level 1 (walks without limitations) to Level 5 (transported in a manual wheelchair). Levels 1 and 2 have almost independent mobility while level 3 can move with assistive devices and levels 4 and 5 are significantly limited and dependent on their helpers for minor movements. A higher score describes worse dysfunction and less dependence during mobility as the level goes up. Gross motor function was categorised as mild (Levels 1 and 2), moderate (level 3) and severe (Levels 4 and 5).

The SDQ is a 25-item questionnaire comprising 5 scales of 5 items each focusing on difficulties relating to emotional functioning, conduct, hyperactivity, and interaction with peers. Scale scores and a total difficulties score (generated by summing the scores from all the scales except the prosocial scale) were documented. Based on the total difficulties score, SDQ scores were categorised into 4 bands: close to average, slightly lower, low, and very low, based on a UK community sample. For 2–4-year-old children, the close to average category contains 80%, the slightly raised category contains 12%, the high category contains 4%, and the very high category contains 4% of the population. For 4-17-year-old parent completed questionnaires, the close to average category contains 80%, the slightly raised category contains 10%, the high category contains 5%, and the very high category contains 5% of the sampled UK population.

The ABAS-II is an instrument used to evaluate adaptive skills that are important to everyday living and assesses three main domains: (i) Conceptual (summarises performance in the following skill areas - communication, functional academics, and self-direction), (ii) Social (leisure and social), and (iii) Practical (community use, home living, health and safety, self-care). The individual response provided for each skill area question was assigned a score. The total score allocated to each domain

was obtained by summing up the skills scores in that domain. Raw scores were converted into composite scores, with a population mean of 100 and a standard deviation of 15, with a lower score signifies worse adaptive behaviour. Composite scores were divided into the following categories based on percentiles (%) of the normative population: very superior > 130 (≥ 98%); superior 120–129 (91–97%); above average 110–119 (75–90%); average 90–109 (25–74%); below average 80–89 (9–24%); borderline 71–79 (3–8%); extremely low 70 or less (≤2%).

Neuropsychological

A blinded neuropsychology assessment was performed at 12 months after randomisation during which cognitive function was assessed using the following age appropriate scales: (i) Bayley Scales of Infant and Toddler Development, third edition (1 to 2 years 5 months); (ii) Wechsler Preschool Primary Scale of Intelligence IV (2 years 6 months to 5 years 11 months), and (iii) Wechsler Intelligence Scale for Children V (6 years to 16 years 11 months).

The Bayley Scales of Infant and Toddler Development (BSID-III) is a widely used and validated measure of cognitive functioning which produces three composite scores: cognitive scale, language scale (receptive and expressive), and motor scale (fine and gross). The Wechsler Preschool Primary Scale of Intelligence IV produces scores for: Verbal Comprehension (VCI), Visual Spatial (VSI), Fluid Reasoning, Working Memory (WMI), Processing Speed (PSI), and Full-scale IQ (FSIQ). The Wechsler Intelligence Scale for Children IV assesses general thinking and reasoning skills and is made up of 10 subtests, yielding 4 composite scores (Verbal Comprehension, Perceptual Reasoning (PRI), Working Memory, and Processing Speed). The Full-Scale IQ (composite score) is an average of these four scales.

Composite standard scores have a mean of 100 and SD of 15. Neurodevelopmental outcome was classified as (i) severe impairment (composite score of <70, >2SD below the mean), (ii) mild impairment (score of 70-84, >1SD below the mean) and (iii) normal neurodevelopmental (score of ≥ 85)

Neuroimmunology

Auto-antibody testing was performed by the clinical neuroimmunology service at the Nuffield Department of Clinical Neurosciences, Oxford.

Neuroimaging

Neuroimaging findings were obtained from clinical CT or MRI scans. In addition, an optional follow up research MRI scan was performed in a subset of participants, where consent was provided. Anonymised scans were analysed for the following:

Initial clinical scan(s):

- Proportion of participants with an abnormal scan
- Distribution of disease – structural and functional anatomy of lesion
- Subset of radiological features (mass effect, hydrocephalus, enhancement, other)

Follow up scan(s)

- Proportion of participants with an abnormal scan
- Lesion resolution/persisting disease
- Presence of new lesions
- Distribution of disease –structural and functional anatomy of lesion
- Subset of radiological features (mass effect, hydrocephalus, enhancement, other).

Supplementary Material Table 1: Baseline neuroimaging results summarising overall findings of acute scans

Participant Number	Type of scan	Overall assessment	Laterality of abnormality
1	MRI	Abnormal	Bilateral
2	MRI	Normal	N/A
3	MRI	Abnormal	Unilateral (Right)
4	CT scan	Normal	N/A
4	MRI	Abnormal	Bilateral
5	MRI	Normal	N/A
6	CT scan	Normal	N/A
6	MRI	Normal	N/A
7	MRI	Not available	Not available

8	MRI	Normal	N/A
8	MRI	Normal	N/A
9	MRI	Abnormal	Bilateral
10	CT scan	Normal	N/A
10	MRI	Abnormal	Bilateral
11	MRI	Normal	N/A
12	CT scan	Normal	N/A
12	CT scan	Normal	N/A
12	MRI	Normal	N/A
13	CT scan	Normal	N/A

Key: N/A= Not applicable

1. Kahwaji J, Barker E, Pepkowitz S, Klapper E, Villicana R, Peng A, et al. Acute hemolysis after high-dose intravenous immunoglobulin therapy in highly HLA sensitized patients. Clin J Am Soc Nephrol. 2009;4(12):1993-7.
