# Supplementary material for: Intravenous immunoglobulin treatment in childhood encephalitis (IgNiTE): a randomised controlled trial
Source: BMJ Open. 2023 Nov 9;13(11):e072134. doi: 10.1136/bmjopen-2023-072134 (PMC10649701; doi:10.1136/bmjopen-2023-072134)
Supplement: Author's manuscript [file bmjopen-2023-072134.draft_revisions.pdf]

BMJ Open is committed to open peer review. As part of this commitment we make the peer review history of every article we publish publicly available.

When an article is published we post the peer reviewers' comments and the authors' responses online. We also post the versions of the paper that were used during peer review. These are the versions that the peer review comments apply to.

The versions of the paper that follow are the versions that were submitted during the peer review process. They are not the versions of record or the final published versions. They should not be cited or distributed as the published version of this manuscript.

BMJ Open is an open access journal and the full, final, typeset and author-corrected version of record of the manuscript is available on our site with no access controls, subscription charges or pay-per-view fees (http://bmjopen.bmj.com).

If you have any questions on BMJ Open's open peer review process please email info.bmjopen@bmj.com

Intravenous immunoglobulin treatment in childhood encephalitis (IgNiTE): A randomised controlled trial

Journal:	*BMJ Open*
Manuscript ID	bmjopen-2023-072134
Article Type:	Original research
Date Submitted by the Author:	24-Jan-2023
Complete List of Authors:	Hill, Matilda; University of Oxford Oxford Vaccine Group, Department of Paediatrics<br>Iro, Mildred; University of Oxford Oxford Vaccine Group<br>Sadarangani, Manish ; University of Oxford Oxford Vaccine Group; University of Oxford Department of Paediatrics<br>Absoud, Michael; Evelina London Children's Hospital Neurosciences Department, Children's Neurosciences; King's College London Faculty of Life Sciences and Medicine<br>Cantrell, Liberty; University of Oxford Oxford Vaccine Group<br>Chong, Kling; UCL Great Ormond Street Institute of Child Health Population Policy and Practice<br>Easton, Ava; Encephalitis Society; University of Liverpool Department of Clinical Infection Microbiology and Immunology<br>Gray, Victoria; Alder Hey Children's NHS Foundation Trust, Clinical Health Psychology<br>Kneen, Rachel; Alder Hey Children's NHS Foundation Trust, Department of Neurology; University of Liverpool, Institute of Infection, Veterinary and Ecological Sciences<br>Lim, Ming; Evelina London Children's Hospital Neurosciences Department, Children's Neurosciences; King's College London Faculty of Life Sciences and Medicine<br>Liu , Xinxue ; University of Oxford Oxford Vaccine Group, Paediatrics<br>Pike, Michael; Oxford University Hospitals NHS Trust, Department of Paediatric Neurology<br>Solomon, Tom; University of Liverpool, Institute of Infection, Veterinary and Ecological Sciences; University of Liverpool, National Institute for Health Research Health Protection Research Unit in Emerging Zoonotic Infections<br>Vincent, Angela; University of Oxford Nuffield Department of Clinical Neurosciences; Weatherall Institute of Molecular Medicine<br>Willis, Louise; University of Oxford Oxford Vaccine Group<br>Yu, Ly-Mee; Oxford University, Department of Primary Care Health Sciences<br>Pollard, Andrew; University of Oxford Oxford Vaccine Group, Paediatrics; University of Oxford Department of Paediatrics
Keywords:	Paediatric neurology < NEUROLOGY, Developmental neurology & neurodisability < PAEDIATRICS, Paediatric neurology < PAEDIATRICS, Paediatric infectious disease & immunisation < PAEDIATRICS, Clinical trials < THERAPEUTICS

For peer review only - http://bmjopen.bmj.com/site/about/guidelines.xhtml

For peer review only - http://bmjopen.bmj.com/site/about/guidelines.xhtml

I, the Submitting Author has the right to grant and does grant on behalf of all authors of the Work (as defined in the below author licence), an exclusive licence and/or a non-exclusive licence for contributions from authors who are: i) UK Crown employees; ii) where BMJ has agreed a CC-BY licence shall apply, and/or iii) in accordance with the terms applicable for US Federal Government officers or employees acting as part of their official duties; on a worldwide, perpetual, irrevocable, royalty-free basis to BMJ Publishing Group Ltd ("BMJ") its licensees and where the relevant Journal is co-owned by BMJ to the co-owners of the Journal, to publish the Work in this journal and any other BMJ products and to exploit all rights, as set out in our licence.

The Submitting Author accepts and understands that any supply made under these terms is made by BMJ to the Submitting Author unless you are acting as an employee on behalf of your employer or a postgraduate student of an affiliated institution which is paying any applicable article publishing charge ("APC") for Open Access articles. Where the Submitting Author wishes to make the Work available on an Open Access basis (and intends to pay the relevant APC), the terms of reuse of such Open Access shall be governed by a Creative Commons licence – details of these licences and which Creative Commons licence will apply to this Work are set out in our licence referred to above.

Other than as permitted in any relevant BMJ Author's Self Archiving Policies, I confirm this Work has not been accepted for publication elsewhere, is not being considered for publication elsewhere and does not duplicate material already published. I confirm all authors consent to publication of this Work and authorise the granting of this licence.

For peer review only

Intravenous immunoglobulin treatment in childhood encephalitis (IgNiTE): A randomised controlled trial

M Hill,[1*] M A Iro,[1*] M Sadarangani,[1,2,3,4] M Absoud,[5,6] L Cantrell[1], W K Chong,[7] C A Clark,[8] A Easton,[9,10] V Gray,[11], R Kneen,[12,13] M Lim,[5,6] X Liu[1], M Pike,[14] T Solomon,[12,15,16,17] A Vincent,[18,19] L Willis,[1] L-M Yu,[20] A J Pollard[1,2], IgNiTE study team

1. Oxford Vaccine Group, Department of Paediatrics, University of Oxford and NIHR Biomedical Research Centre, Oxford University Hospitals NHS Foundation Trust, Oxford, UK
2. Department of Paediatrics, Oxford University Hospitals NHS Foundation Trust, Oxford, UK
3. Vaccine Evaluation Center, BC Children's Hospital Research Institute, University of British Columbia, Vancouver BC, Canada
4. Department of Pediatrics, University of British Columbia, Vancouver, British Columbia, Canada
5. Department of Children's Neurosciences, Evelina London Children's Hospital at Guy's and St Thomas' NHS Foundation Trust, King's Health Partners Academic Health Science Centre, London, UK
6. Department of Women and Children's Health, Faculty of Life Sciences and Medicine, King's College London, London, UK
7. UCL Great Ormond Street Institute of Child Health, London, UK
8. Institute of Child Health, University College London, London, UK
9. The Encephalitis Society, Malton, North Yorkshire, UK
10. Department of Clinical Infection, Microbiology and Immunology, University of Liverpool, UK
11. Clinical Health Psychology, Alder Hey Children's NHS Foundation Trust, Liverpool, UK
12. Institute of Infection, Veterinary and Ecological Sciences, University of Liverpool, Liverpool, UK
13. Department of Neurology, Alder Hey Children's NHS Foundation Trust, Liverpool, UK
14. Department of Paediatric Neurology, Oxford University Hospitals NHS Trust, Oxford, UK
15. National Institute for Health Research Health Protection Research Unit in Emerging and Zoonotic Infections, University of Liverpool, Liverpool, UK
16. Walton Centre NHS Foundation Trust, Liverpool, UK
17. The Pandemic Institute, Liverpool, UK
18. Nuffield Department of Clinical Neurosciences, University of Oxford, Oxford, UK
19. Weatherall Institute of Molecular Medicine, University of Oxford, Oxford, UK
20. Nuffield Department of Primary Care Health Sciences, University of Oxford, Oxford, UK

*M Hill and M A Iro contributed equally to this paper.

Corresponding author:
Dr Matilda Hill
matilda.hill@paediatrics.ox.ac.uk
Oxford Vaccine Group, Department of Paediatrics, University of Oxford and NIHR Biomedical Research Centre, Oxford University Hospitals NHS Foundation Trust, Oxford, UK OX3 7LE.
01865 611400

Word count
Main text (from Introduction to Conclusion): 4,091

Keywords
'Childhood encephalitis'; 'Encephalitides'; 'Autoimmune encephalitis'; 'Immune-mediated'; 'Brain inflammation'; 'Encephalitis treatment'; 'IVIG'; '; 'Epilepsy'; 'Neurological outcome'; 'GOS-E Peds'

Abstract

Objective: To investigate whether intravenous immunoglobulin (IVIG) improves neurological outcomes in children with encephalitis when administered early in the illness.

For peer review only - http://bmjopen.bmj.com/site/about/guidelines.xhtml

Design: Phase 3b multi-centre, double-blind, randomised placebo-controlled trial.

Setting: Twenty-one hospitals in the UK.

Participants: Children aged 6 months to 16 years with a diagnosis of acute or sub-acute encephalitis.

Intervention: Two doses (1g/kg/dose) of either IVIG or matching placebo given 24-36 hours apart, in addition to standard treatment.

Main outcome measure: The primary outcome was a ‘good recovery’ at 12 months after randomisation, defined as a score of ≤2 on the Paediatric Glasgow Outcome Score Extended (GOS-E Peds).

Secondary outcome measures: The secondary outcomes were clinical, neurological, neuroimaging and neuropsychological results, identification of the proportion of children with immune-mediated encephalitis, and IVIG safety data.

Results: 18 participants were randomised to receive either IVIG (n=10) or placebo (n=8) between 23 December 2015 and 26 September 2017. The study was terminated early due to slow recruitment, and therefore did not reach the pre-determined sample size required to achieve the primary study objective, thus the results are descriptive. At 12 months after randomisation, nine of the 18 participants (IVIG n=5/10 (50%), placebo n=4/8 (50%)) made a good recovery and five participants (IVIG n=3/10 (30%), placebo n=2/8 (25%)) made a poor recovery. Three participants (IVIG n=1/10 (10%), placebo n=2/8 (25%)) had a new diagnosis of epilepsy during the study period. Two participants were found to have specific autoantibodies associated with autoimmune encephalitis. No serious adverse events were reported in participants receiving IVIG.

Conclusions: IgNiTE study findings support existing evidence of poor neurological outcomes in children with encephalitis. However, the study was halted prematurely due to slow recruitment, and was therefore underpowered to evaluate the effect of early IVIG treatment compared to placebo in childhood encephalitis.

Trial registration: Clinical Trials.gov NCT02308982; ICRCTN registry ISRCTN15791925

Article Summary

Strengths and limitations of this study

- This was the first ever multicentre, randomised controlled trial evaluating IVIG treatment for all-cause encephalitis in children.
- The study had clinically meaningful endpoints and was run to a very high standard, with rigorous blinding procedures throughout.
- The primary objective could not be evaluated due to early withdrawal of funding following slower than expected recruitment, resulting in the predefined sample size not being achieved.
- Lessons learned from this study are valuable and should be considered for future studies evaluating treatments for childhood encephalitis.

Introduction

For peer review only

Encephalitis is a major cause of illness and death globally(1-3). It is characterised by inflammation of the brain parenchyma causing neurological dysfunction which manifests acutely as altered mental state and can have long-term sequalae including neurological disability and seizures. In children, the most common causes of encephalitis are autoimmune disorders and infections, with viral encephalitis being the most frequently identified aetiology(4-10). It often takes time to reach a definitive diagnosis, and a cause may not be found despite extensive investigation in at least one fifth of children(4, 6, 7, 10).

Encephalitis is more prevalent among children than adults, with an estimated incidence of 4.0 – 12.6 per 100,000 person years for children in high income countries (7, 10-14). There is a substantially higher burden of childhood encephalitis in regions such as southeast Asia where the Japanese encephalitis virus is endemic(2, 8). Childhood encephalitis carries a significant mortality rate; this ranges from 5%-13%, dependent on setting and aetiology(4, 8, 10, 15, 16). Approximately half of children who survive an episode of encephalitis will have long term sequalae which may include neurological deficits, physical disability, cognitive impairment, neuropsychiatric disorders, and epilepsy(4, 8, 10, 15, 17-19). Childhood encephalitis is therefore associated with a high global economic, healthcare, and social burden(1, 3, 8, 15, 20).

Whilst there is good evidence for the efficacy of aciclovir in the management of encephalitis caused by herpes simplex virus (HSV) and varicella zoster virus (VZV)(21, 22), there are limited therapeutic options for other types of childhood encephalitis and the mainstay of treatment is supportive care. Treatment strategies for autoimmune encephalitis include methylprednisolone, plasma exchange and intravenous immunoglobulin, but the recommendations for their use are based largely on studies in individuals with specific types of autoimmune encephalitis, retrospective cohort studies and expert opinion(23-26). Furthermore, these therapies are often only implemented after a definitive autoimmune cause for encephalitis has been identified or all alternative diagnoses, including infectious, have been ruled out.

IVIG is used successfully in other inflammatory and neurological conditions in children(27, 28), however, there have been no high-quality studies to support or refute its use in children with all types of encephalitis (29, 30). Inflammation of the brain parenchyma is the common cause of altered neurological function in encephalitis, regardless of the aetiology, and it may therefore be postulated that interventions which attenuate the inflammation early in the illness are likely to have the greatest efficacy in reducing the severity of the acute illness, mortality and neurological sequalae of childhood encephalitis.

In this study, we set out to establish if early IVIG treatment, in addition to standard care, improves outcomes for children with encephalitis of all aetiologies.

Methods

Study Design

IgNiTE was a randomised, double blinded, parallel arm, placebo-controlled study to compare early IVIG treatment with placebo in the treatment of childhood encephalitis in individuals aged 6 months to 16 years. It was conducted across 21 National Health Service (NHS) hospitals in the UK. Participants were followed up for 12 months after randomisation, with outcomes assessed during the acute admission, at 4-8 weeks after discharge from acute care, at 6 months after randomisation, and 12 months after randomisation.

The trial was prospectively registered with ClinicalTrials.gov (identifier NCT 02308982) on 5 December 2014. The trial was assigned an International Standard Randomised Controlled Trial Number on 24 June 2015 (ISRCTN 15791925), and a European Clinical Trials

For peer review only - http://bmjopen.bmj.com/site/about/guidelines.xhtml

Database number (2014-002997-35). A Trial Steering Committee (TSC) was established to oversee the trial, and an independent Data Monitoring and Ethics Committee (DMEC) was set up to monitor the safety, efficacy, and overall conduct of the study.

The original trial protocol was published on 03 November 2016(31). The protocol was amended after the early termination of the trial to remove endpoints which could not be derived from the data collected and to update the statistical analysis section; the amended protocol is available in the supplementary material.

Participants

Eligible participants were hospitalised children aged between 6 weeks and 16 years who met the case definition for possible encephalitis based on the International Encephalitis Consensus(32), where written informed consent was obtained from parents or guardians, and assent was given if appropriate.

Exclusion criteria were a high clinical suspicion of bacterial meningitis; prior receipt of IVIG during the admission or known contraindication to IVIG; traumatic brain injury; history of metabolic encephalopathy; stroke, toxic or hypertensive encephalopathy; pre-existing demyelinating disorder; significant renal impairment; hypercoagulable state; hyperprolinaemia; participation in another research trial involving an immunomodulatory treatment; pregnancy; any significant disease or disorder which may put the participants at risk because of participation in the trial, influence the result of the trial, or the participant's ability to participate in the trial; involvement in another research trial involving an investigational medicinal product which has potential immunomodulatory or neuroprotective effects.

Intervention

Two doses of 1g/kg/dose of either IVIG or a matching volume of placebo were given 24-36 hours apart, with the first dose administered as soon as possible after enrolment and within five working days from the suspicion of an encephalitis diagnosis.

The active treatment (IVIG) used in the study was Privigen (100mg/ml solution), manufactured and provided by CSL Behring. The placebo was a mixture of 0.9% saline and 0.1% human albumin solution, manufactured at the Royal Broadgreen and Liverpool Aseptic Production Unit, Liverpool, UK under cGMP conditions and its Manufacturer's Importer's Authorisation (IMP) licence.

Randomisation and blinding

Participants were randomised 1:1 to IVIG or placebo treatment after consent was obtained. Randomisation was stratified by age group (< 1 year, 1-4 years, 5-9 years, 10-14 years and ≥15 years) and steroid treatment at the time of randomisation, using stratified block randomisation with randomly varying block sizes. Randomisation was performed using a secure web-based randomisation system (Sortition®) which was developed by the Clinical Trials Unit in the Nuffield Department of Primary Care Health Sciences, University of Oxford.

Participants, their parents or guardians, clinical staff, and all study staff (including staff involved in recruitment, administration of study treatment, data collection and entry, and laboratory analyses) were blind to the treatment allocation through the entire study period. Study monitors who were independent of the study and all site pharmacists were unblinded to ensure dispensing of the correct allocation and robust investigational medicinal product (IMP) management at each study site. The placebo and IVIG were visually identical, due to the additional of 0.1% human albumin solution to 0.9% in the placebo.

For peer review only - http://bmjopen.bmj.com/site/about/guidelines.xhtml

Primary Outcome

The primary outcome was good recovery, which was defined as a score of 2 or less on the paediatric version of the Glasgow Outcome Score Extended (GOS-E Peds) at 12 months after randomisation.

The GOS-E Peds is based on the GOS-E, a gold standard for measuring outcomes in adults with traumatic brain injury. It is a validated tool for use in children, and provides a developmentally appropriate structured interview necessary to evaluate children across different age groups(33). Participants were assigned a GOS-E Peds score: 1-Upper Good Recovery, 2-Lower Good Recovery, 3-Upper Moderate Disability, 4-Lower Moderate Disability, 5-Upper Severe Disability, 6-Lower Severe Disability, 7-Vegetative State, and 8-Death. 'Good recovery' was defined as a GOS-E Peds score of ≤ 2, and a score of >2 indicated 'poor recovery'.

Secondary outcomes

Secondary clinical outcomes included admission to intensive care unit, requirement for invasive ventilation, length of acute hospital stay, new diagnoses of epilepsy in the 12 months after randomisation and need for anti-epileptic treatment.

Secondary neurological and functional outcomes comprised GOS-E Peds assessment at 6 months after randomisation, and Liverpool Outcome Score (LOS) assessment, Pediatric Quality of Life Score (PedsQL) assessment, Gross Motor Function and Classification System (GMFCS) assessment, Strengths and Difficulty Questionnaire (SDQ) assessment and Adaptive Behavior Assessment System – second edition (ABAS-II) assessment at 4-8 weeks after discharge from acute care and at 12 months after randomisation.

Secondary neuropsychological outcomes were cognitive assessment at 12 months after randomisation using the age-appropriate scales: (i) Bayley Scales of Infant and Toddler Development, third edition (1 to 2 years 5 months); (ii) Wechsler Preschool Primary Scale of Intelligence IV (2 years 6 months to 5 years 11 months), and (iii) Wechsler Intelligence Scale for Children V (6 years to 16 years 11 months).

The secondary neuroimmunology outcome was identification of autoantibodies. The antibodies tested for were for antibodies against live neurons, Aquaporin 4 (AQP4), N-Methyl-D-aspartate receptor, Myelin oligodendrocyte glycoprotein (MOG), leucine-rich, glioma inactivated 1 (LGI1), and Contactin-associated protein-like 2 (CASPR2).

Secondary neuroimaging outcomes comprised assessment of CT or MRI brain scans performed as part of routine care during the acute illness, and follow up scans performed at 6 months after randomisation in a subset of participants, where consent was provided.

Secondary safety outcomes included safety data obtained throughout the study, and a full blood count performed for all participants 24-48 hours following the second dose of the study treatment to monitor for haemolysis which has previously been described with high concentrations of IVIG treatment(34). Safety data comprised adverse events (AEs) and adverse events of special interest (AESIs) occurring in the first five days following receipt of each dose of the study drug, serious adverse events (SAEs) occurring up until 6 months after randomisation and serious adverse reactions (SARs) occurring throughout the study period. Information on any deaths occurring up to 12 months after randomisation was also collected.

Further information regarding to the secondary outcomes is provided in the Supplementary material.

Protocol Amendments

For peer review only

The IgNiTE study was halted in October 2017 after the withdrawal of funding due to slower than anticipated recruitment, despite proposed alternative strategies to deliver on the study objectives, including a revision of the recruitment timeline to ensure that this important clinical study could report. Where possible, follow up activities were completed for all participants who were already enrolled into the trial, as per the protocol. The protocol was amended to remove endpoints which could not be derived from the data collected and to update the statistical analysis section.

Statistical analysis

A sample size of 308 participants recruited over a 24 month period (154 per group, with approximate 10% attrition rate) was planned to achieve 90% power (at 5% level of significance) to detect at least a 20% clinically significant treatment difference from 43% in the 'good recovery' rate (defined as a GOS-E Peds score of ≤ 2) by 12 months after randomisation. This was based on the results of a large observational study on autoimmune encephalitis(26).

At the time the trial was halted, only 18 participants had been recruited. The trial was therefore underpowered to perform hypothesis testing of outcomes, subgroup comparisons or sensitivity analyses. Therefore, all analyses performed were descriptive. The analyses were performed on the intention-to-treat population this included all 18 participants who were randomised. In the analysis of the adverse events, the population analysed were the 16 participants who received study treatment.

Patient and public involvement (PPI)

The Encephalitis Society was involved in the planning of this study, and the training of research nurses and study recruiters. A representative of The Encephalitis Society was on the Trial Management Group and provided a patient-centred research perspective to the study design and conduct. PPI groups were consulted in the development of the essential documents for the study including the participant information sheet and consent forms. Three PPI representatives with previous personal experiences of encephalitis sat on the Trial Steering Committee (TSC) and contributed to providing overall oversight of the study. Study update meetings were held to which patients previously affected by encephalitis were invited to share their experiences with the study team.

Results

Participants

A total of 884 patients were screened for eligibility between 23rd December 2015 and 26th September 2017 across 21 NHS hospitals, of whom 18 participants were enrolled and randomised. 10 participants were assigned to IVIG treatment, and 8 patients were assigned to placebo. The study flow diagram is shown in Figure 1.

Table 1 summarises the baseline characteristics of participants by treatment arm. The mean age of the participants was 4.09 years (interquartile range (IQR) 2.0 – 11.8), 44% were male, and 89% were of White ethnicity.

For peer review only - http://bmjopen.bmj.com/site/about/guidelines.xhtml

Baseline characteristic		IVIG (n=10)	Placebo (n=8)	All (n=18)
**Age at randomisation (years)**	Median (IQR)	5.55 (1.52, 11.8)	4.09 (2.71, 9.64)	4.09 (2.0, 11.8)
**Sex**	Male	4 (40%)	4 (50%)	8 (44.4%)
	Female	6 (60%)	4 (50%)	10 (55.6%)
**Ethnicity**	White	8 (80%)	8 (100%)	16 (88.9%)
	Asian	1 (10%)	0 (0%)	1 (5.6%)
	Missing	1 (10%)	0 (0%)	1 (5.6%)
**History of immunocompromise**	No	9 (90%)	7 (87.5%)	16 (88.9%)
	Missing	1 (10%)	1 (12.5%)	2 (11.1%)
**Previous diagnosis of encephalitis**	No	9 (90%)	7 (87.5%)	16 (88.9%)
	Missing	1 (10%)	1 (12.5%)	2 (11.1%)
**History of encephalopathic illness**	No	9 (90%)	7 (87.5%)	16 (88.9%)
	Missing	1 (10%)	1 (12.5%)	2 (11.1%)
**Pre-existing diagnosis of epilepsy**	No	9 (90%)	7 (87.5%)	16 (88.9%)
	Missing	1 (10%)	1 (12.5%)	2 (11.1%)

Table 1: Baseline characteristics of enrolled participants

Primary Outcome

At 12 months after randomisation, nine participants (50%; IVIG n=5/10 [50%]; placebo n=4/8 [50%]) made a good recovery, defined as a GOS-E Peds score of ≤ 2. Five participants (28%; IVIG n=3/10 [30%], placebo n=2/8 [25%]) made a poor recovery, defined as a GOS-E Peds score of >2. Four participants (22%; IVIG n=2/10 [20%], placebo n=2/8 [25%]) did not undergo a GOS-E Peds assessment at this timepoint. Table 2 displays these results.

	IVIG (N = 10)	Placebo (N = 8)	Overall N = 18
**GOSE-Peds Score**			
1.Upper good recovery	4 (40%)	4 (50%)	8 (44%)
2.Lower good recovery	1 (10%)	0 (0%)	1 (6%)
5.Upper severe disability	1 (10%)	1 (13%)	2 (11)
6.Lower severe disability	2 (20%)	1 (13%)	3 (17%)
Participants with missing data due to being withdrawn or lost to follow up	2 (20%)	2 (25%)	4 (22%)

Table 2: GOS-E Peds scores at 12 months after randomisation

For peer review only - http://bmjopen.bmj.com/site/about/guidelines.xhtml

Secondary Outcomes

Clinical Outcomes
Ten participants (56%; IVIG n=5/10 [50%], placebo=5/8 [63%]) were admitted to intensive care during their acute admission with encephalitis, as shown in Table 3. Nine of these participants (90%; IVIG n=4/5 [80%], placebo n=5/5 [100%]) required invasive ventilation, for a median duration of 2 days (IQR 2.0 – 3.0). The median length of stay on intensive care was 4.5 days (IQR 3.0 - 6.8) and the overall median length of hospitalisation for acute care was 11 days (IQR 7.8 - 19.5).

Three participants (17%; IVIG n=1/10 [10%], placebo n=2/8 [25%]) had a new diagnosis of epilepsy during the study period. Five participants (28%; IVIG n= 2/10 [20%], placebo n=3/8 [38%]) had incomplete data for this outcome.

**Outcome**		**IVIG (N = 10)**	**Placebo (N = 8)**	**Overall (N = 18)**
**During hospital stay**				
Duration of ventilation	Median (IQR)	2.5 (2.0, 3.5) [n = 4]	2.0 (2.0, 3.0) [n = 5]	2.0 (2.0, 3.0) [n = 9]
Length of ICU stay	Median (IQR)	4.0 (3.0, 6.0) [n = 5]	5.0 (2.0, 10.0) [n = 5]	4.5 (3.0, 6.8) [n = 10]
Length of hospitalisation for acute care	Median (IQR)	12.0 (8.0, 27.0) [n = 9]	8.0 (6.5, 14.0) [n = 7]	11.0 (7.8, 19.5) [n = 16]
**6 months post randomisation**				
New diagnosis of epilepsy since discharge	n (%)	1 (10%)	1 (13%)	2 (11%)
Anti-epileptic treatment since discharge	n (%)	1 (10%)	1 (13%)	2 (11%)
**12 months post randomisation**				
New diagnosis of epilepsy since discharge	n (%)	0 (0%)	1 (13%)	1 (6%)
Anti-epileptic treatment since discharge	n (%)	0 (0%)	0 (0%)	0 (0%)

Table 3: Secondary clinical outcomes

GOS-E Peds assessment at 6 months
Fifteen participants underwent GOS-E Peds assessment at 6 months after randomisation. Eight participants (44%; IVIG n=4/10 [40%], placebo n= 4/8 [50%]) made a good recovery and seven participants (39%; IVIG n=4/10 [50%], placebo n=3/8 [38%]) made a poor recovery at this timepoint, as shown in Table 4.

Liverpool Outcome Score (LOS)
Fifteen participants had a LOS assessment at 4-8 weeks after discharge from acute care. Five participants (28%; IVIG n=3/10 [30%], placebo n=2/8 [25%]) made a full recovery, defined as a LOS of >4. Ten participants (56%; IVIG n= 5/10 [50%], placebo n=5/8 [63%]) had minor to severe sequelae. Table 4 displays the breakdown of these results.

For peer review only - http://bmjopen.bmj.com/site/about/guidelines.xhtml

Fourteen participants had a LOS assessment at 12 months after randomisation. Six participants (33%; IVIG n= 4/10 [40%], placebo n=2/8 [25%]) had made full recovery at this timepoint, and eight participants (44%; IVIG n=4/10 [40%], placebo n=4/8 [50%]) had minor to severe sequelae.

Paediatric Quality of Life Assessment (PedsQL)
Seven participants (39%; IVIG n=5/10 [50%], placebo n=2/8 [25%]) had a PedsQL assessment at 4-8 weeks after discharge from acute care, and eight participants (44%; IVIG n=6/10 [60%], placebo n=2/8 [25%]) had a PedsQL assessment at 12 months after randomisation. At 4-8 weeks after discharge from acute care, the mean PedsQL score was 77.9 (standard deviation (SD) 11.10) and 56.5 (SD 7.8) for the IVIG and placebo group, respectively. At 12 months, mean PedsQL scores were 79.9 (SD 21.6) and 63.7 (SD 30.1) for the IVIG and placebo groups, respectively. This data is displayed in Table 4.

Gross Motor and Function Classification System (GMFCS)
Seven participants underwent a GMFCS assessment at 4-8 weeks after discharge from acute care, and eight participants underwent assessment at 12 months after randomisation. At 4-8 weeks after discharge, all seven participants assessed (39%; IVIG n=5/10 [50%]; placebo n=2/8 [25%]) had mild impairment of gross motor functioning. At 12 months after randomisation, all eight participants (44%; IVIG n=6/10 [60%]; placebo n=2/8 [25%]) experienced mild or severe impairment of gross motor function, as demonstrated by Table 4.

Strengths and difficulties assessment (SDQ)
SDQ results were available for seven participants (39%; IVIG n=5/10 [50%], placebo n=2/8 [25%]) at 4-8 weeks after discharge from acute care and eight participants (44%; IVIG n=6/10 [60%], placebo n=2/8 [25%]) at 12 months after randomisation.

At 4-8 week after discharge from acute care, five participants (28%; IVIG n=4 [40%]; placebo n=1 [13%]) had a close to average SDQ score, one participant (6%; IVIG n=1/10 [10%]) had a slightly raised SDQ score and one participant (6%; placebo n=1/8 [13%]) had a very high SDQ score. At 12 months after randomisation, the same number of participants had a close to average score and slightly raised score, but two participants (11%; IVIG n=1/10 [10%], placebo n=1/8 [13%]) had a very high SDQ score.

Adaptive Behaviour Assessment System – Second Edition (ABAS-II)
Eight participants had an ABAS-II assessment at 4-8 weeks after discharge from acute care, and seven participants had an ABAS-II assessment at 12 months after randomisation (see Table 4). At 4-8 weeks after discharge, five participants (28%; IVIG n=4/10 [40%], placebo n=1/8 [13%]) had an ABAS-II score that was either similar or higher than the average score of the normative population, and three participants (17%; IVIG n=2/10 [20%], placebo n=1/8 [13%]) had a score that was lower than the average score. At 12 months after randomisation, the same number of participants had a score that was below the average at 12 months after randomisation, but four participants (22%; IVIG n=3/10 [30%], placebo n=1/8 [13%]) had a score that was either similar or higher than the average score at this timepoint.

For peer review only

Outcome	4-8 weeks post discharge		12 months post randomisation	
	IVIG (N=10)	Placebo (N=8)	IVIG (N=10)	Placebo (N=8)
**LOS**				
2.Severe sequelae	2 (20%)	2 (25%)	2 (20%)	2 (25%)
3.Moderate sequelae	2 (20%)	3 (38%)	1 (10%)	1 (13%)
4.Minor sequelae	1 (10%)	0 (0%)	1 (10%)	1 (3%)
5.Full recovery	3 (30%)	2 (25%)	4 (40%)	2 (25%)
Missing data due to withdrawal or loss to follow up of participant	1 (10%)	1 (13%)	2 (20%)	2 (25%)
Missing data – assessment not performed	1 (10%)	0 (0%)	0 (%)	0 (%)
**PedsQL**				
Mean (SD)	77.9 (11.1)	56.5 (7.8)	79.9 (21.6)	63.7 (30.1)
Missing data due to withdrawal or loss to follow up of participant	1 (10%)	1 (13%)	2 (20%)	2 (25%)
Missing data – assessment not performed	4 (40%)	5 (63%)	2 (20%)	4 (50%)
**SDQ**				
Close to average	4 (40%)	1 (13%)	4 (40%)	1 (13%)
Slightly raised	1 (10%)	0 (0%)	1 (10%)	0 (0%)
Very high	0 (0%)	1 (13%)	1 (10%)	1 (13%)
Missing data due to withdrawal or loss to follow up of participant	1 (10%)	1 (13%)	2 (20%)	2 (25%)
Missing data – assessment not performed	4 (40%)	5 (63%)	2 (20%)	4 (50%)
**ABAS**				
Very Superior	0 (0%)	0 (0%)	1 (10%)	0 (0%)
Superior	1 (10%)	0 (0%)	1 (10%)	0 (0%)
Above average	1 (10%)	0 (0%)	1 (10%)	0 (0%)
Average	2 (20%)	1 (13%)	0 (0%)	1 (13%)
Below average	0 (0%)	1 (13%)	1 (10%)	0 (0%)
Borderline	1 (10%)	0 (0%)	0 (0%)	0 (0%)
Extremely low	1 (10%)	0 (0%)	1 (10%)	1 (13%)
Missing data due to withdrawal or loss to follow up of participant	1 (10%)	1 (13%)	2 (20%)	2 (25%)
Missing data – assessment not performed	3 (30%)	5 (63%)	3 (30%)	4 (50%)
**GMFCS***				
Mild	5 (50%)	2 (25%)	6 (60%)	1 (13%)
Severe			0 (0%)	1 (13%)
Missing data due to withdrawal or loss to follow up of participant	1 (10%)	1 (13%)	2 (20%)	2 (25%)
Missing data – assessment not performed	4 (40%)	5 (63%)	2 (20%)	4 (50%)
**GOSE-Peds at 6 months post randomisation**				
	**IVIG (N=10)**	**Placebo (N=8)**		
1.Upper good recovery	4 (40%)	4 (50%)		
3.Upper moderate disability	1 (10%)	1 (13%)		
5.Upper severe disability	0 (0%)	1 (13%)		
6.Lower severe disability	3 (30%)	1 (13%)		
Missing data due to withdrawal or loss to follow up of participant	2 (20%)	1 (13%)		

For peer review only

Missing data – assessment not performed	0 (0%)	0 (0%)

Table 4: Secondary neurological and functional outcomes

Neuropsychology Outcomes
Thirteen participants (72%; IVIG n=8/10 [80%]; placebo n=5/8 [63%]) had blinded neuropsychology assessment at 12 months after randomisation. Four of these participants (30%; IVIG n =2/8 [25%], placebo n=2/5 [40%]) were unable to complete the full battery of assessments due to attentional or behavioural needs.

Five participants (28%; IVIG n=4/10 [40%], placebo n=1/8 [13%]) had a score of ≥ 85 (indicating normal development) for full scale IQ (FSIQ), six (33%; IVIG n=4/10 [40%]; placebo n=2/8 [25%]) for verbal comprehension (VCI), five (28%; IVIG n=4/10 [40%], placebo n=1/8 [13%]) for visual spatial (VSI); four (22%; IVIG n=4/10 [40%]) for working memory (WMI); and four (22%; IVIG n=3/10 [30%]; placebo n=1/8 [13%]) for perceptual reasoning (PRI). Two participants (IVIG n=1, placebo n=1) were assessed using the Bayley scale of infant development, one participant (IVIG n=1) had severe neurodevelopmental impairment while the other (placebo n=1) had a normal neurodevelopmental outcome. These results are displayed in Table 5.

Participant	Age at Assessment	Bayley cognitive score	FSIQ	VCI	VSI/PRI	WMI	PSI
**Placebo arm**							
1	4y 8m	-	*	*	*	*	*
2	5y 6m	-	79	95	79	75	71
3	2y 10m	-	*	*	*	*	*
4	2y 0m	110	-	-	-	-	-
5	16y 10m	-	89	99	88	83	94
**IVIG arm**							
6	4y 5m	-	*	*	*	*	*
7	9y 2m	-	104	92	111	107	116
8	14y 1m	-	95	102	90	99	91
9	8y 8m	-	88	93	96	91	83
10	2y 2m	55	-	-	-	-	-
11	3y 9m	-	65	60	75	72	-
12	2y 1m	-	*	*	*	*	*
13	14y 6m	-	119	108	110	110	131

Key: Green = normal neurodevelopmental score, Yellow = mild impairment, Red = severe impairment. *Young person unable to complete full battery due to attention or behavioural needs

Table 5: Neuropsychology outcomes at 12 months after randomisation

Neuroimaging Outcomes
Nineteen acute neuroimaging scans were available for 13 participants (72%; IVIG n=8/10 [80%], placebo n=5/8 [63%]). Five of these scans (for five unique participants; IVIG n=2/8 [25%], placebo n=3/8 [38%]) had abnormal findings; all of these were MRI scans (see Supplementary Material Table 1). Four of the abnormal scans showed bilateral lesions.

For peer review only - http://bmjopen.bmj.com/site/about/guidelines.xhtml

There were nine follow up scans for eight unique participants (IVIG n=5/10 [50%], placebo n=3/8 [50%]); six of these scans (for five unique participants; IVIG n=3/5 [60%], placebo n=1/4 [25%]) were normal and unchanged from the acute scan. Three follow up scans (for three unique participants; IVIG n=2/5 [40%], placebo n=1/3 [33%]) had abnormal findings; two of these were unchanged from the acute scans and an acute scan was not available for comparison one participant.

Autoantibody Testing
Twelve participants (67%; IVIG n=7/10 [70%], placebo n=5/8 [63%]) had autoantibody testing. One participant (placebo n=1) was positive for LGI1 antibodies, and one participant (placebo n=1) was positive for MOG antibodies. Two additional participants (IVIG n=2) were positive for IgG binding to the surface of live neurons, indicating the presence of IgG antibodies binding to neurons, but negative for antibodies to the specific antigens tested, , indicating the presence of undefined IgG antibodies that could be pathogenic.

Safety Data
Ten serious adverse events occurred in three participants in the placebo group and none in the IVIG group. None of the SAEs were judged to be related to the study treatment. One participant in the IVIG group reported an adverse event of special interest; the participant developed a fever during the IVIG infusion, however, this was judged to be unrelated to the study treatment. None of the participants experienced haemolysis following receipt of two doses of study treatment. No deaths occurred during the study period.

Discussion

The IgNiTE study was terminated early due to slower than expected recruitment and was therefore unable to provide conclusive evidence regarding the efficacy of IVIG in the treatment of childhood encephalitis. Thus, it remains unknown whether early administration of IVIG in children with encephalitis offers clinical benefit, irrespective of the underlying aetiology.

Whilst the IgNITE study was unable to address the primary study objective, the results do provide evidence of the poor outcomes experiences by many children with encephalitis. Almost a third of participants made a poor recovery based on GOS-E Peds assessment at 12 months after randomisation. Other measures of neurological outcomes consistently demonstrated a heavy burden of disability; 44% of patients had minor to severe sequalae at 12 months according to the LOS assessment, and the same proportion of patients experienced mild or severe impairment of gross motor function at the same timepoint. The proportion of children with functional impairments on the SDQ and ABAS-II assessments at 12 months after randomisation was lower, but this was likely due to fewer participants completing these assessments.

The results also demonstrate the heavy burden childhood encephalitis places on healthcare. Over half of participants required admission to intensive care during the acute illness, and 90% of these children were intubated. The overall median length of acute hospital care for participants was 11 days, compared with a mean length of hospital stay of 1.64 days for children and young people following an emergency admission in the UK(35). Furthermore, many children with encephalitis likely require ongoing non-acute hospital care for neurorehabilitation, as evidenced by the high proportion of participants with lasting disability.

These data are consistent with previous studies of childhood encephalitis in high-income settings. In a prospective Australian study involving 287 children with encephalitis, 49% of children required admission to intensive care, median length of hospitalisation was 11 days and 27% of children had moderate to severe neurodisability at hospital discharge(4). Of

For peer review only - http://bmjopen.bmj.com/site/about/guidelines.xhtml

note, they used the adult Glasgow Outcome Score (GOS) tool for assessment of outcomes and did not capture children with mild to moderate neurodisability, which may explain the lower proportion of children with reported neurodisability compared with the IgNiTE study. A meta-analysis of long-term outcomes of childhood encephalitis reported 47% of children to have long-term sequalae in studies in high income countries, although there was no standardised definition of sequalae used across these studies(17).

Limitations of the study

The main limitation of the IgNiTE study is that the predefined sample size was not met, and the primary study objective was therefore not achieved. The study initially planned to recruit 308 participants over a 24 month period, based on the results of a large observational study on autoimmune encephalitis(26). However, recruitment to the study was slower than expected; the majority of patients assessed for eligibility did not meet the inclusion criteria, suggesting that the use of stringest diagnostic criteria may have precluded the inclusion of some children with clinically suspected encephalitis..

Of the patients who met the inclusion criteria, the main reasons for exclusion were prior or planned IVIG treatment as part of routine care, and study timeline restrictions. The use of IVIG as part of routine care demonstrates that some clinicians were already convinced of the benefit of IVIG in childhood encephalitis despite the lack of high quality evidence and the fact that IVIG was not commissioned for use in childhood encephalitis at the time the study was running. This highlights the challenges of conducting randomised controlled trials when treating clinicians are not in clinical equipoise.

The requirement for parents or guardians to provide informed consent at an exquisitely sensitive time for the family also impacted recruitment; two-thirds of eligible participants declined enrolment. Other factors which may have contributed to the low consent rate include the limited time frame for enrolment, the blinded nature of the trial, and the trial duration and practicalities, including the requirement for study visits and additional blood sampling(36).

Lessons learned and future research

Further research is required to establish whether early IVIG is of therapeutic benefit in the treatment of childhood encephalitis, irrespective of the underlying aetiology. The IgNiTE study demonstrated the feasibility of conducting a randomised controlled trial to investigate this important question, and highlighted the need for a longer recruitment period to reach a definitive answer. Future studies should anticipate the recruitment challenges discussed above and consider strategies such as incorporating a pilot phase, using less strict inclusion criteria, allowing a wider time frame in which participants can be enrolled, and adopting approaches to optimise consent rates in eligible patients.

Conclusion

The IgNiTE study was terminated prematurely due to slow recruitment and therefore did not reach the pre-determined sample size required to evaluate the effect of IVIG compared to placebo in childhood encephalitis.. However, the study results support existing evidence of poor neurological outcomes in many children with encephalitis. This provides further compelling evidence of the need for better treatments in childhood encephalitis. Future studies are required to establish if treatment with IVIG is of benefit in children with encephalitis of all causes. Such studies should take into account the challenges encountered and lessons learnt from the IgNiTE study.

For peer review only - http://bmjopen.bmj.com/site/about/guidelines.xhtml

What is already known on this topic

- Childhood encephalitis is associated with poor outcomes for many children
- There are limited treatment options for children with encephalitis
- The benefit of IVIG in children with all types of encephalitis is unclear

What this study adds

- The study provides supportive evidence of the poor long term neurological and functional outcomes for many children with encephalitis
- The study was unable to answer the question of whether IVIG improves outcomes for children with encephalitis, but there are important lessons to learnt for future studies investigating this question

Ethics Statements

The study was approved by the UK National Research Ethics Service (NRES) committee (South Central—Oxford A; REC 14/SC/1416). Written approval from local Research and Development (R&D) departments at each participating site was obtained before recruitment was commenced at each site.
This study was conducted in accordance with the Declaration of Helsinki (1996), in full conformity with the International Conference on Harmonisation of Technical Requirements for Registration of Pharmaceuticals for Human Use (ICH) Guidelines for Good Clinical Practice (CPMP/ICH/135/95 July 1996), the Research Governance Framework, and the Medicines for Human Use (Clinical Trial) Regulations 2004.

Funding statement

This project was funded by the National Institute for Health Research (NIHR) Efficacy and Mechanism Evaluation programme (grant number 12/212/15). The investigational medicinal product (IMP), intravenous immunoglobulin (Privigen) was provided by CSL Behring.

Data sharing statement

All data requests should be submitted to the corresponding author for consideration. Access to anonymised data may be granted following review.

Acknowledgements

The authors would like to thank all the participants, families, and health-care professionals for their involvement in the study.

Author Statement

Authors's individual contributions

The trial was conceptualised by MP and AJP, and designed with input from ML, MA, MS, MAI, L-MY, RK and TS. MP, MS, RK, TS, WKC, CAC, ML, MA, AV, VG, AE, L-MY and AJP are named investigators on the IgNiTE trial. Each co-author provided specific additional contributions within their area of expertise; paediatric neurology (MP, RK, ML, MA), paediatric infectious diseases (MS, AJP), paediatric neuropsychology (VG), neuroimaging (CAC, WKC), neuroimmunology (AV), statistics (L-MY, LC, XL) and patient group (AE). MAI is the lead doctor for the trial and LW is the lead nurse for the trial. MH prepared the first version of the manuscript. All authors contributed to the manuscript and have approved the final manuscript for publication.

The IgNiTE Study Team

For peer review only - http://bmjopen.bmj.com/site/about/guidelines.xhtml

For peer review only

Trial Management Group
Prof Sir Andrew J Pollard (Chief Investigator), Dr Ming Lim, Prof Tom Solomon, Dr Rachel Kneen, Dr Michael Absoud, Dr Mike Pike, Dr Manish Sadarangani, Dr Kling Chong, Dr Chris A. Clark, Dr Victoria Gray

Trial coordinating team (Oxford)
Dr Mildred Iro (Clinical ResearchFfellow), Louise Willis (Research Nurse/Project Manager), David Kerr, Sophie Bradshaw, Svetlana Milca, Simon Kerridge, Emma Plested, Yama Mujadidi

IMP management
Shakeel Herwitker, Mandy Wan

Trial Steering Committee
Dr Claire Cameron, Dr Ming Lim, Dr Adilia Warris, Dr Federico Martinon-Torres, Mike Bale, Sonia Bale, Alan Percival, and Dr Ava Easton

Data Monitoring and Ethics Committee
Dr Charles Warlow (Chairperson), Dr Jo Haviland (statistician), Dr David Pace and Dr Simon Nadel

Statistics team
Dr Ly-Mee Yu, Dr Meryn Voysey, Dr Xinxue Liu, Liberty Cantrell

Laboratory team (Oxford)
Sagida Bibi, Amy Beveridge, Amber Thompson, Daniel O'Connor, Prof Sarosh Irani, Dr Patrick Waters

Neuropsychological assessment support
Lauren Burke, Victoria Gray

Oxford Vaccine Group
Emma Plested, Parvinder Alley

Recruitment sites

**Name of hospital**	**Principal Investigator**
Oxford University Hospitals	*Andrew Pollard*
Great Ormond Street Hospital	*Sanjay Bhate*
Alder Hey Children's Hospital	*Rachel Kneen*
St George's Hospital	*Paul Heath*
Barts and the London (Royal London)	*Anna Riddell*
Guy's and St Thomas's - Evelina	*Ming Lim*
Sheffield Children's Hospital	*Archana Desurkar*
NHS Grampian	*Elma Stephen*
Heart of England	*Steve Welch*
Pennine - North Manchester Children's Hospital	*Paddy McMaster*
Ninewells (Tayside Health Board)	*Alice Jollands*
Royal Hospital for Sick Children, Edinburgh	*Jay Shetty*
University Hospitals Bristol	*Kayal Vijayakumar*
Imperial – St Mary's Hospital	*Leena Mewasingh*
Nottingham University Hospitals	*William Whitehouse*
Hull Royal Infirmary	*Vishal Mehta*

For peer review only - http://bmjopen.bmj.com/site/about/guidelines.xhtml

University Hospitals of North Midland	*John Alexander*
Leeds Teaching Hospital	*John Livingston*
Royal Preston Hospital	*Christian de Goede*
York Teaching Hospital	*Dominic Smith*
Royal Cornwall Hospitals	*Andrew Collinson*

Declared competing interests of authors

Mildred Iro was a trainee member on the NIHR Efficacy and Mechanism Evaluation Programme Funding Committee from October 2020 to October 2021. Michael Absoud has received a grant from the NIHR in the last 36 months, for research unrelated to the submitted work. MS has been an investigator on projects funded by GlaxoSmithKline, Merck, Moderna, Pfizer, Sanofi-Pasteur, Seqirus, Symvivo and VBI Vaccines; all funds have been paid to his institute. Ava Easton is Chief Executive of the Encephalitis Society, which has previously received grants from CSL Behring (UK). Ming Lim has received grants from the GOSH charity, Boston Children's Hospital Research Fund and Action Medical Research in the last 36 months, all for research unrelated to the submitted work. Ming Lim is co-chair of the European Paediatric Neurology Education and Training Board and works for an institution which holds research accounts with Roche (Switzerland), Octapharma (Switzerland) and Novartis (Switzerland). Tom Solomon is supported by the NIHR Health Protection Research Unit in Emerging and Zoonotic Infections, NIHR Programme Grant for Applied Research, NIHR Global Health Research on Brain Infections and the European Union's Horizon 2020 research and innovation program ZikaPLAN. Tom Solomon is a consultant for the MHRA Vaccine Benefit Risk Expert Working Group. Angela Vincent is a consultant for Aspen NewCo Inc and has received honoraria from UCB and Alexion. Ly-Mee Yu was a member of the NIHR Health Technology Assessment Efficient Study Designs from November 2015 to July 2016. Andrew Pollard is chair of the Department of Health and Social Care's Joint committee on Vaccines and Immunisation (JCVI) and was a member of WHO's SAGE until 1st January 2022. Oxford University has entered a partnership with AstraZenenca on COVID-19 vaccines, but Andrew Pollard does not participate in the JCVI COVID-19 committee.

References

1. Tyler KL. Acute Viral Encephalitis. N Engl J Med. 2018;379(6):557-66.
2. Wang H, Zhao S, Wang S, Zheng Y, Wang S, Chen H, et al. Global magnitude of encephalitis burden and its evolving pattern over the past 30 years. J Infect. 2022;84(6):777-87.
3. Dr Julia Granerod AE, Dr Nicholas Davies, Dr Benedict Michael, Professor Tom Solomon, Dr Ava Easton. Encephalitis: an in-depth review and gap analysis of key variables affecting global disease burden. Encephalitis Society; 2022 July 21 2022.
4. Britton PN, Dale RC, Blyth CC, Clark JE, Crawford N, Marshall H, et al. Causes and Clinical Features of Childhood Encephalitis: A Multicenter, Prospective Cohort Study. Clin Infect Dis. 2020;70(12):2517-26.
5. Timothy A. Erickson EM, Flor M. Munoz, Timothy Lotze, Rodrigo Hasbun, Eric Brown,Kristy O. Murray, . Infectious and Autoimmune Causes of Encephalitis in Children. Pediatrics. 2020;145 (6).
6. de Blauw D, Bruning AHL, Busch CBE, Kolodziej LM, Jansen NJG, van Woensel JBM, et al. Epidemiology and Etiology of Severe Childhood Encephalitis in The Netherlands. Pediatr Infect Dis J. 2020;39(4):267-72.
7. George BP, Schneider EB, Venkatesan A. Encephalitis hospitalization rates and inpatient mortality in the United States, 2000-2010. PloS one. 2014;9(9):e104169.

For peer review only

8. Pommier JD, Gorman C, Crabol Y, Bleakley K, Sothy H, Santy K, et al. Childhood encephalitis in the Greater Mekong region (the SouthEast Asia Encephalitis Project): a multicentre prospective study. Lancet Glob Health. 2022;10(7):e989-e1002.
9. Granerod J, Crowcroft NS. The epidemiology of acute encephalitis. Neuropsychol Rehabil. 2007;17(4-5):406-28.
10. Wickström R, Fowler Å, Bogdanovic G, Bennet R, Eriksson M. Review of the aetiology, diagnostics and outcomes of childhood encephalitis from 1970 to 2009. Acta Paediatr. 2017;106(3):463-9.
11. Britton PN, Khoury L, Booy R, Wood N, Jones CA. Encephalitis in Australian children: contemporary trends in hospitalisation. Arch Dis Child. 2016;101(1):51-6.
12. Jmor F, Emsley HC, Fischer M, Solomon T, Lewthwaite P. The incidence of acute encephalitis syndrome in Western industrialised and tropical countries. Virol J. 2008;5:134.
13. Iro MA, Sadarangani M, Goldacre R, Nickless A, Pollard AJ, Goldacre MJ. 30-year trends in admission rates for encephalitis in children in England and effect of improved diagnostics and measles-mumps-rubella vaccination: a population-based observational study. Lancet Infect Dis. 2017;17(4):422-30.
14. Rantakallio P, Leskinen M, von Wendt L. Incidence and prognosis of central nervous system infections in a birth cohort of 12,000 children. Scand J Infect Dis. 1986;18(4):287-94.
15. Griffiths MJ, Lemon JV, Rayamajhi A, Poudel P, Shrestha P, Srivastav V, et al. The functional, social and economic impact of acute encephalitis syndrome in Nepal--a longitudinal follow-up study. PLoS Negl Trop Dis. 2013;7(9):e2383.
16. Granerod J, Ambrose HE, Davies NW, Clewley JP, Walsh AL, Morgan D, et al. Causes of encephalitis and differences in their clinical presentations in England: a multicentre, population-based prospective study. Lancet Infect Dis. 2010;10(12):835-44.
17. Khandaker G, Jung J, Britton PN, King C, Yin JK, Jones CA. Long-term outcomes of infective encephalitis in children: a systematic review and meta-analysis. Dev Med Child Neurol. 2016;58(11):1108-15.
18. Fowler A, Stödberg T, Eriksson M, Wickström R. Childhood encephalitis in Sweden: etiology, clinical presentation and outcome. Eur J Paediatr Neurol. 2008;12(6):484-90.
19. Fowler Å, Stödberg T, Eriksson M, Wickström R. Long-term Outcomes of Acute Encephalitis in Childhood. Pediatrics. 2010;126(4):e828-e35.
20. Deng X, Yan R, Li ZQ, Tang XW, Zhou Y, He H. Economic and disease burden of Japanese encephalitis in Zhejiang Province, 2013-2018. PLoS Negl Trop Dis. 2021;15(6):e0009505.
21. Sköldenberg B, Alestig K, Burman L, Forkman A, Lövgren K, Norrby R, et al. ACYCLOVIR VERSUS VIDARABINE IN HERPES SIMPLEX ENCEPHALITIS: Randomised Multicentre Study in Consecutive Swedish Patients. The Lancet. 1984;324(8405):707-11.
22. Whitley RJ, Alford CA, Hirsch MS, Schooley RT, Luby JP, Aoki FY, et al. Vidarabine versus acyclovir therapy in herpes simplex encephalitis. N Engl J Med. 1986;314(3):144-9.
23. Zuliani L, Nosadini M, Gastaldi M, Spatola M, Iorio R, Zoccarato M, et al. Management of antibody-mediated autoimmune encephalitis in adults and children: literature review and consensus-based practical recommendations. Neurol Sci. 2019;40(10):2017-30.
24. Dubey D, Britton J, McKeon A, Gadoth A, Zekeridou A, Lopez Chiriboga SA, et al. Randomized Placebo-Controlled Trial of Intravenous Immunoglobulin in Autoimmune LGI1/CASPR2 Epilepsy. Ann Neurol. 2020;87(2):313-23.

For peer review only - http://bmjopen.bmj.com/site/about/guidelines.xhtml

25. Nosadini M, Eyre M, Molteni E, Thomas T, Irani SR, Dalmau J, et al. Use and Safety of Immunotherapeutic Management of N-Methyl-d-Aspartate Receptor Antibody Encephalitis: A Meta-analysis. JAMA Neurol. 2021;78(11):1333-44.
26. Titulaer MJ, McCracken L, Gabilondo I, Armangué T, Glaser C, Iizuka T, et al. Treatment and prognostic factors for long-term outcome in patients with anti-NMDA receptor encephalitis: an observational cohort study. Lancet Neurol. 2013;12(2):157-65.
27. Oates-Whitehead RM, Baumer JH, Haines L, Love S, Maconochie IK, Gupta A, et al. Intravenous immunoglobulin for the treatment of Kawasaki disease in children. Cochrane Database Syst Rev. 2003;2003(4):CD004000.
28. Hughes RA, Swan AV, van Doorn PA. Intravenous immunoglobulin for Guillain-Barre syndrome. Cochrane Database Syst Rev. 2014;2014(9):CD002063.
29. Iro MA, Martin NG, Absoud M, Pollard AJ. Intravenous immunoglobulin for the treatment of childhood encephalitis. Cochrane Database Syst Rev. 2017;10:CD011367.
30. Wagner JN, Leibetseder A, Troescher A, Panholzer J, von Oertzen TJ. Efficacy and safety of intravenous immunoglobulins for the treatment of viral encephalitis: a systematic literature review. J Neurol. 2022;269(2):712-24.
31. Iro MA, Sadarangani M, Absoud M, Chong WK, Clark CA, Easton A, et al. ImmunoglobuliN in the Treatment of Encephalitis (IgNiTE): protocol for a multicentre randomised controlled trial. BMJ Open. 2016;6(11):e012356.
32. Venkatesan A, Tunkel AR, Bloch KC, Lauring AS, Sejvar J, Bitnun A, et al. Case definitions, diagnostic algorithms, and priorities in encephalitis: consensus statement of the international encephalitis consortium. Clin Infect Dis. 2013;57(8):1114-28.
33. Beers SR, Wisniewski SR, Garcia-Filion P, Tian Y, Hahner T, Berger RP, et al. Validity of a pediatric version of the Glasgow Outcome Scale-Extended. J Neurotrauma. 2012;29(6):1126-39.
34. Kahwaji J, Barker E, Pepkowitz S, Klapper E, Villicana R, Peng A, et al. Acute hemolysis after high-dose intravenous immunoglobulin therapy in highly HLA sensitized patients. Clin J Am Soc Nephrol. 2009;4(12):1993-7.
35. Kossarova EsKaL. Focus on: Emergency hospital care for children and young people. QualityWatch; 2017 April 2017.
36. V Shilling PW, H Hickey, E Sowden,, RL Smyth BY. Processes in recruitment to randomised
controlled trials of medicines for
children (RECRUIT): a qualitative study. Health Technology Assessment. 2011;15.

For peer review only

Figures

Figure 1: Study flow diagram

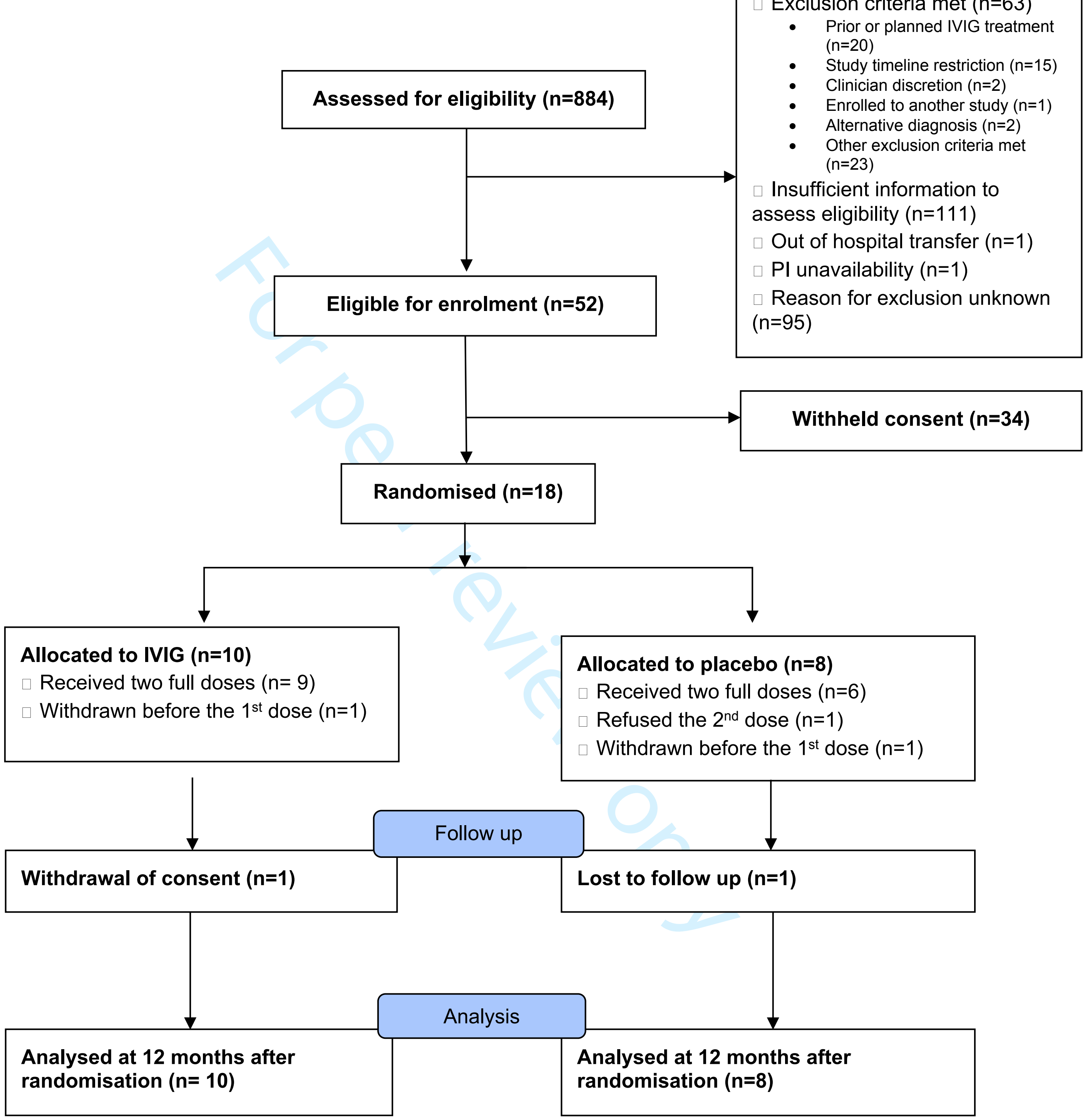

Supplementary Material

Secondary outcomes

Clinical

Secondary clinical outcomes were obtained from routinely collected medical information. These comprised admission to intensive care unit, invasive ventilation requirement, and length of hospital stay, defined as the number of days from admission to a recruiting hospital to discharge from acute care (i.e. not including days in hospital for neurorehabilitation). At 6 and 12 months after randomisation, information on new diagnosis of epilepsy and need for anti-epileptic treatment since discharge were collected.

Neurological and Functional

Secondary neurological outcomes were assessed using age appropriate questionnaires and outcome scores which comprised the GOS-E Peds (assessed at 6 months after randomisation), Liverpool Outcome Score (LOS), Pediatric Quality of Life Score (PedsQL), Gross Motor Function and Classification System (GMFCS), Strengths and Difficulty Questionnaire (SDQ), and Adaptive Behaviour Assessment System, second edition (ABAS-II), all assessed at 4-8 weeks after discharge from acute care and 12 months after randomisation.

The LOS is a validated tool for assessing level of disability after encephalitis in infants and children. It was originally designed to assess disease burden following JE and its use has been extended to other forms of encephalitis. For each participant, a total score (sum of scores for all questions) and an outcome score (the lowest score for any single question) were documented. Based on the outcome score only, participants were assigned to one of 5 outcome categories: 5-Full recovery, 4-Minor sequelae, 3-Moderate sequelae, 2-Severe sequelae, and 1-Death. 'Good recovery' was defined as a LOS of 5 and a score of ≤ 4 indicated 'poor recovery'.

The PedsQL is a brief measure of health-related quality of life comprised of 23 items assessing quality of life in 4 domains: physical functioning (8 items), emotional functioning (5 items), social functioning (5 items) and school functioning (5 items). Based on the scores in each domain, two summary scores (physical health and

For peer review only

psychosocial health summary scores) as well as a total scale score were computed. Total scale scores are presented. A higher total scale score indicates better quality of life.

The GMFCS is an assessment tool based on self-initiated movement and assesses motor function in three areas - walking, sitting, and standing. It uses 5 levels to describe the motor function limitations, taking into consideration age, the use of mobility aids and the quality of movement and is rated from Level 1 (walks without limitations) to Level 5 (transported in a manual wheelchair). Levels 1 and 2 have almost independent mobility while level 3 can move with assistive devices and levels 4 and 5 are significantly limited and dependent on their helpers for minor movements. A higher score describes worse dysfunction and less dependence during mobility as the level goes up. Gross motor function was categorised as mild (Levels 1 and 2), moderate (level 3) and severe (Levels 4 and 5).

The SDQ is a 25-item questionnaire comprising 5 scales of 5 items each focusing on difficulties relating to emotional functioning, conduct, hyperactivity, and interaction with peers. Scale scores and a total difficulties score (generated by summing the scores from all the scales except the prosocial scale) were documented. Based on the total difficulties score, SDQ scores were categorised into 4 bands: close to average, slightly lower, low, and very low, based on a UK community sample. For 2–4-year-old children, the close to average category contains 80%, the slightly raised category contains 12%, the high category contains 4%, and the very high category contains 4% of the population. For 4-17-year-old parent completed questionnaires, the close to average category contains 80%, the slightly raised category contains 10%, the high category contains 5%, and the very high category contains 5% of the sampled UK population.

The ABAS-II is an instrument used to evaluate adaptive skills that are important to everyday living and assesses three main domains: (i) Conceptual (summarises performance in the following skill areas - communication, functional academics, and self-direction), (ii) Social (leisure and social), and (iii) Practical (community use, home living, health and safety, self-care). The individual response provided for each skill area question was assigned a score. The total score allocated to each domain

For peer review only

was obtained by summing up the skills scores in that domain. Raw scores were converted into composite scores, with a population mean of 100 and a standard deviation of 15, with a lower score signifies worse adaptive behaviour. Composite scores were divided into the following categories based on percentiles (%) of the normative population: very superior > 130 (≥ 98%); superior 120–129 (91–97%); above average 110–119 (75–90%); average 90–109 (25–74%); below average 80–89 (9–24%); borderline 71–79 (3–8%); extremely low 70 or less (≤2%).

Neuropsychological

A blinded neuropsychology assessment was performed at 12 months after randomisation during which cognitive function was assessed using the following age appropriate scales: (i) Bayley Scales of Infant and Toddler Development, third edition (1 to 2 years 5 months); (ii) Wechsler Preschool Primary Scale of Intelligence IV (2 years 6 months to 5 years 11 months), and (iii) Wechsler Intelligence Scale for Children V (6 years to 16 years 11 months).

The Bayley Scales of Infant and Toddler Development (BSID-III) is a widely used and validated measure of cognitive functioning which produces three composite scores: cognitive scale, language scale (receptive and expressive), and motor scale (fine and gross). The Wechsler Preschool Primary Scale of Intelligence IV produces scores for: Verbal Comprehension (VCI), Visual Spatial (VSI), Fluid Reasoning, Working Memory (WMI), Processing Speed (PSI), and Full-scale IQ (FSIQ). The Wechsler Intelligence Scale for Children IV assesses general thinking and reasoning skills and is made up of 10 subtests, yielding 4 composite scores (Verbal Comprehension, Perceptual Reasoning (PRI), Working Memory, and Processing Speed). The Full-Scale IQ (composite score) is an average of these four scales.

Composite standard scores have a mean of 100 and SD of 15. Neurodevelopmental outcome was classified as (i) severe impairment (composite score of <70, >2SD below the mean), (ii) mild impairment (score of 70-84, >1SD below the mean) and (iii) normal neurodevelopmental (score of ≥ 85)

Neuroimmunology

For peer review only

Auto-antibody testing was performed by the clinical neuroimmunology service at the Nuffield Department of Clinical Neurosciences, Oxford.

Neuroimaging

Neuroimaging findings were obtained from clinical CT or MRI scans. In addition, an optional follow up research MRI scan was performed in a subset of participants, where consent was provided. Anonymised scans were analysed for the following:

Initial clinical scan(s):

- Proportion of participants with an abnormal scan
- Distribution of disease – structural and functional anatomy of lesion
- Subset of radiological features (mass effect, hydrocephalus, enhancement, other)

Follow up scan(s)

- Proportion of participants with an abnormal scan
- Lesion resolution/persisting disease
- Presence of new lesions
- Distribution of disease –structural and functional anatomy of lesion
- Subset of radiological features (mass effect, hydrocephalus, enhancement, other).

Supplementary Material Table 1: Baseline neuroimaging results summarising overall findings of acute scans

Participant Number	Age at time of acute scan	Type of scan	Overall assessment	Laterality of abnormality
1	3 years 7 months	MRI	Abnormal	Bilateral
2	14 years	MRI	Normal	N/A
3	1 year 9 months	MRI	Abnormal	Unilateral (Right)
4	13 years	CT scan	Normal	N/A
4	13 years	MRI	Abnormal	Bilateral
5	8 years 2 months	MRI	Normal	N/A
6	15 years 9 months	CT scan	Normal	N/A
6	15 years 9 months	MRI	Normal	N/A
7	1 year	MRI	Not available	Not available

For peer review only

8	2 years 8 months	MRI	Normal	N/A
8	2 years 8 months	MRI	Normal	N/A
9	4 years 6 months	MRI	Abnormal	Bilateral
10	7 years 8 months	CT scan	Normal	N/A
10	7 years 8 months	MRI	Abnormal	Bilateral
11	7 years 9 months	MRI	Normal	N/A
12	1 year	CT scan	Normal	N/A
12	1 year	CT scan	Normal	N/A
12	1 year	MRI	Normal	N/A
13	1 year 1 month	CT scan	Normal	N/A

Key: N/A= Not applicable

1. Kahwaji J, Barker E, Pepkowitz S, Klapper E, Villicana R, Peng A, et al. Acute hemolysis after high-dose intravenous immunoglobulin therapy in highly HLA sensitized patients. Clin J Am Soc Nephrol. 2009;4(12):1993-7.

For peer review only

CONSORT 2010 checklist of information to include when reporting a randomised trial*

Section/Topic	Item No	Checklist item	Reported on page No
**Title and abstract**			
	1a	Identification as a randomised trial in the title	1
	1b	Structured summary of trial design, methods, results, and conclusions (for specific guidance see CONSORT for abstracts)	2-3
**Introduction**			
Background and objectives	2a	Scientific background and explanation of rationale	3-4
	2b	Specific objectives or hypotheses	4
**Methods**			
Trial design	3a	Description of trial design (such as parallel, factorial) including allocation ratio	4
	3b	Important changes to methods after trial commencement (such as eligibility criteria), with reasons	6
Participants	4a	Eligibility criteria for participants	4
	4b	Settings and locations where the data were collected	4
Interventions	5	The interventions for each group with sufficient details to allow replication, including how and when they were actually administered	5
Outcomes	6a	Completely defined pre-specified primary and secondary outcome measures, including how and when they were assessed	5,6
	6b	Any changes to trial outcomes after the trial commenced, with reasons	6
Sample size	7a	How sample size was determined	6
	7b	When applicable, explanation of any interim analyses and stopping guidelines	-
Randomisation:			
Sequence generation	8a	Method used to generate the random allocation sequence	5
	8b	Type of randomisation; details of any restriction (such as blocking and block size)	5
Allocation concealment mechanism	9	Mechanism used to implement the random allocation sequence (such as sequentially numbered containers), describing any steps taken to conceal the sequence until interventions were assigned	5
Implementation	10	Who generated the random allocation sequence, who enrolled participants, and who assigned participants to interventions	5

For peer review only

Blinding	11a	If done, who was blinded after assignment to interventions (for example, participants, care providers, those assessing outcomes) and how	5
	11b	If relevant, description of the similarity of interventions	5
Statistical methods	12a	Statistical methods used to compare groups for primary and secondary outcomes	6
	12b	Methods for additional analyses, such as subgroup analyses and adjusted analyses	6
**Results**			
Participant flow (a diagram is strongly recommended)	13a	For each group, the numbers of participants who were randomly assigned, received intended treatment, and were analysed for the primary outcome	7
	13b	For each group, losses and exclusions after randomisation, together with reasons	7
Recruitment	14a	Dates defining the periods of recruitment and follow-up	7
	14b	Why the trial ended or was stopped	9
Baseline data	15	A table showing baseline demographic and clinical characteristics for each group	Table 1
Numbers analysed	16	For each group, number of participants (denominator) included in each analysis and whether the analysis was by original assigned groups	Figure 1
Outcomes and estimation	17a	For each primary and secondary outcome, results for each group, and the estimated effect size and its precision (such as 95% confidence interval)	Tables 2-5. Pages 7-9
	17b	For binary outcomes, presentation of both absolute and relative effect sizes is recommended	N/A
Ancillary analyses	18	Results of any other analyses performed, including subgroup analyses and adjusted analyses, distinguishing pre-specified from exploratory	N/A
Harms	19	All important harms or unintended effects in each group (for specific guidance see CONSORT for harms)	9
**Discussion**			
Limitations	20	Trial limitations, addressing sources of potential bias, imprecision, and, if relevant, multiplicity of analyses	10
Generalisability	21	Generalisability (external validity, applicability) of the trial findings	10,11
Interpretation	22	Interpretation consistent with results, balancing benefits and harms, and considering other relevant evidence	11
**Other information**			
Registration	23	Registration number and name of trial registry	1
Protocol	24	Where the full trial protocol can be accessed, if available	Supplementary material
Funding	25	Sources of funding and other support (such as supply of drugs), role of funders	11

For peer review only - http://bmjopen.bmj.com/site/about/guidelines.xhtml

*We strongly recommend reading this statement in conjunction with the CONSORT 2010 Explanation and Elaboration for important clarifications on all the items. If relevant, we also recommend reading CONSORT extensions for cluster randomised trials, non-inferiority and equivalence trials, non-pharmacological treatments, herbal interventions, and pragmatic trials. Additional extensions are forthcoming: for those and for up to date references relevant to this checklist, see www.consort-statement.org.

For peer review only

BMJ Open

Intravenous immunoglobulin treatment in childhood encephalitis (IgNiTE): A randomised controlled trial

Journal:	*BMJ Open*
Manuscript ID	bmjopen-2023-072134.R1
Article Type:	Original research
Date Submitted by the Author:	24-Jul-2023
Complete List of Authors:	Hill, Matilda; University of Oxford Oxford Vaccine Group, Department of Paediatrics<br>Iro, Mildred; University of Oxford Oxford Vaccine Group<br>Sadarangani, Manish ; University of Oxford Oxford Vaccine Group; University of Oxford Department of Paediatrics<br>Absoud, Michael; Evelina London Children's Hospital Neurosciences Department, Children's Neurosciences; King's College London Faculty of Life Sciences and Medicine<br>Cantrell, Liberty; University of Oxford Oxford Vaccine Group<br>Chong, Kling; UCL Great Ormond Street Institute of Child Health Population Policy and Practice<br>Clark, Christopher; Institute of Child Health, University College London<br>Easton, Ava; Encephalitis Society; University of Liverpool Department of Clinical Infection Microbiology and Immunology<br>Gray, Victoria; Alder Hey Children's NHS Foundation Trust, Clinical Health Psychology<br>Kneen, Rachel; Alder Hey Children's NHS Foundation Trust, Department of Neurology; University of Liverpool, Institute of Infection, Veterinary and Ecological Sciences<br>Lim, Ming; Evelina London Children's Hospital Neurosciences Department, Children's Neurosciences; King's College London Faculty of Life Sciences and Medicine<br>Liu , Xinxue ; University of Oxford Oxford Vaccine Group, Paediatrics<br>Pike, Michael; Oxford University Hospitals NHS Trust, Department of Paediatric Neurology<br>Solomon, Tom; University of Liverpool, Institute of Infection, Veterinary and Ecological Sciences; University of Liverpool, National Institute for Health Research Health Protection Research Unit in Emerging Zoonotic Infections<br>Vincent, Angela; University of Oxford Nuffield Department of Clinical Neurosciences; Weatherall Institute of Molecular Medicine<br>Willis, Louise; University of Oxford Oxford Vaccine Group<br>Yu, Ly-Mee; Oxford University, Department of Primary Care Health Sciences<br>Pollard, Andrew; University of Oxford Oxford Vaccine Group, Paediatrics; University of Oxford Department of Paediatrics<br>The, IgNiTE study team; University of Oxford
**Primary Subject Heading**:	Paediatrics

For peer review only - http://bmjopen.bmj.com/site/about/guidelines.xhtml

Secondary Subject Heading:	Infectious diseases, Pharmacology and therapeutics, Neurology, Immunology (including allergy)
Keywords:	Paediatric neurology < NEUROLOGY, Developmental neurology & neurodisability < PAEDIATRICS, Paediatric neurology < PAEDIATRICS, Paediatric infectious disease & immunisation < PAEDIATRICS, Clinical trials < THERAPEUTICS

SCHOLARONE™ Manuscripts

I, the Submitting Author has the right to grant and does grant on behalf of all authors of the Work (as defined in the below author licence), an exclusive licence and/or a non-exclusive licence for contributions from authors who are: i) UK Crown employees; ii) where BMJ has agreed a CC-BY licence shall apply, and/or iii) in accordance with the terms applicable for US Federal Government officers or employees acting as part of their official duties; on a worldwide, perpetual, irrevocable, royalty-free basis to BMJ Publishing Group Ltd ("BMJ") its licensees and where the relevant Journal is co-owned by BMJ to the co-owners of the Journal, to publish the Work in this journal and any other BMJ products and to exploit all rights, as set out in our licence.

The Submitting Author accepts and understands that any supply made under these terms is made by BMJ to the Submitting Author unless you are acting as an employee on behalf of your employer or a postgraduate student of an affiliated institution which is paying any applicable article publishing charge ("APC") for Open Access articles. Where the Submitting Author wishes to make the Work available on an Open Access basis (and intends to pay the relevant APC), the terms of reuse of such Open Access shall be governed by a Creative Commons licence – details of these licences and which Creative Commons licence will apply to this Work are set out in our licence referred to above.

Other than as permitted in any relevant BMJ Author's Self Archiving Policies, I confirm this Work has not been accepted for publication elsewhere, is not being considered for publication elsewhere and does not duplicate material already published. I confirm all authors consent to publication of this Work and authorise the granting of this licence.

For peer review only

Intravenous immunoglobulin treatment in childhood encephalitis (IgNiTE): A randomised controlled trial

M Hill,[1*] M A Iro,[1*] M Sadarangani,[1,2,3,4] M Absoud,[5,6] L Cantrell[1], W K Chong,[7] C A Clark,[8] A Easton,[9,10] V Gray,[11], R Kneen,[12,13] M Lim,[5,6] X Liu[1], M Pike,[14] T Solomon,[12,15,16,17] A Vincent,[18,19] L Willis,[1] L-M Yu,[20] A J Pollard[1,2], IgNiTE study team

1. Oxford Vaccine Group, Department of Paediatrics, University of Oxford and NIHR Biomedical Research Centre, Oxford University Hospitals NHS Foundation Trust, Oxford, UK
2. Department of Paediatrics, Oxford University Hospitals NHS Foundation Trust, Oxford, UK
3. Vaccine Evaluation Center, BC Children's Hospital Research Institute, University of British Columbia, Vancouver BC, Canada
4. Department of Pediatrics, University of British Columbia, Vancouver, British Columbia, Canada
5. Department of Children's Neurosciences, Evelina London Children's Hospital at Guy's and St Thomas' NHS Foundation Trust, King's Health Partners Academic Health Science Centre, London, UK
6. Department of Women and Children's Health, Faculty of Life Sciences and Medicine, King's College London, London, UK
7. UCL Great Ormond Street Institute of Child Health, London, UK
8. Institute of Child Health, University College London, London, UK
9. The Encephalitis Society, Malton, North Yorkshire, UK
10. Department of Clinical Infection, Microbiology and Immunology, University of Liverpool, UK
11. Clinical Health Psychology, Alder Hey Children's NHS Foundation Trust, Liverpool, UK
12. Institute of Infection, Veterinary and Ecological Sciences, University of Liverpool, Liverpool, UK
13. Department of Neurology, Alder Hey Children's NHS Foundation Trust, Liverpool, UK
14. Department of Paediatric Neurology, Oxford University Hospitals NHS Trust, Oxford, UK
15. National Institute for Health Research Health Protection Research Unit in Emerging and Zoonotic Infections, University of Liverpool, Liverpool, UK
16. Walton Centre NHS Foundation Trust, Liverpool, UK
17. The Pandemic Institute, Liverpool, UK
18. Nuffield Department of Clinical Neurosciences, University of Oxford, Oxford, UK
19. Weatherall Institute of Molecular Medicine, University of Oxford, Oxford, UK
20. Nuffield Department of Primary Care Health Sciences, University of Oxford, Oxford, UK

*M Hill and M A Iro contributed equally to this paper.

Corresponding author:
Dr Matilda Hill
matilda.hill@paediatrics.ox.ac.uk
Oxford Vaccine Group, Department of Paediatrics, University of Oxford and NIHR Biomedical Research Centre, Oxford University Hospitals NHS Foundation Trust, Oxford, UK OX3 7LE.
01865 611400

Word count
Main text (from Introduction to Conclusion): 5,189

Keywords
'Childhood encephalitis'; 'Encephalitides'; 'Autoimmune encephalitis'; 'Immune-mediated'; 'Brain inflammation'; 'Encephalitis treatment'; 'IVIG'; '; 'Epilepsy'; 'Neurological outcome'; 'GOS-E Peds'

Abstract

Objective: To investigate whether intravenous immunoglobulin (IVIG) improves neurological outcomes in children with encephalitis when administered early in the illness.

For peer review only - http://bmjopen.bmj.com/site/about/guidelines.xhtml

Design: Phase 3b multi-centre, double-blind, randomised placebo-controlled trial.

Setting: Twenty-one hospitals in the UK.

Participants: Children aged 6 months to 16 years with a diagnosis of acute or sub-acute encephalitis, with a planned sample size of 308.

Intervention: Two doses (1g/kg/dose) of either IVIG or matching placebo given 24-36 hours apart, in addition to standard treatment.

Main outcome measure: The primary outcome was a 'good recovery' at 12 months after randomisation, defined as a score of ≤2 on the Paediatric Glasgow Outcome Score Extended (GOS-E Peds).

Secondary outcome measures: The secondary outcomes were clinical, neurological, neuroimaging and neuropsychological results, identification of the proportion of children with immune-mediated encephalitis, and IVIG safety data.

Results: 18 participants were recruited from 12 hospitals and randomised to receive either IVIG (n=10) or placebo (n=8) between 23 December 2015 and 26 September 2017. The study was terminated early following withdrawal of funding due to slower than anticipated recruitment, and therefore did not reach the pre-determined sample size required to achieve the primary study objective, thus the results are descriptive. At 12 months after randomisation, nine of the 18 participants (IVIG n=5/10 (50%), placebo n=4/8 (50%)) made a good recovery and five participants (IVIG n=3/10 (30%), placebo n=2/8 (25%)) made a poor recovery. Three participants (IVIG n=1/10 (10%), placebo n=2/8 (25%)) had a new diagnosis of epilepsy during the study period. Two participants were found to have specific autoantibodies associated with autoimmune encephalitis. No serious adverse events were reported in participants receiving IVIG.

Conclusions: The IgNiTE study findings support existing evidence of poor neurological outcomes in children with encephalitis. However, the study was halted prematurely and was therefore underpowered to evaluate the effect of early IVIG treatment compared to placebo in childhood encephalitis.

Trial registration: Clinical Trials.gov NCT02308982; ICRCTN registry ISRCTN15791925

Strengths and limitations of this study

- This was the first ever multicentre, randomised controlled trial evaluating IVIG treatment for all-cause encephalitis in children.
- The study had clinically meaningful endpoints and was run to a very high standard, with rigorous blinding procedures throughout.
- Recruitment to the study was limited by the strict inclusion and exclusion criteria, the limited time window for enrolment, and lack of equipoise amongst clinicans.

Introduction

Encephalitis is a major cause of illness and death globally(1-3). It is characterised by inflammation of the brain parenchyma causing neurological dysfunction which manifests acutely as altered mental state and can have long-term sequalae including neurological disability and seizures. In children, the most common causes of encephalitis are autoimmune disorders and infections, with viral encephalitis being the most frequently

For peer review only

identified aetiology(4-10). It often takes time to reach a definitive diagnosis, and a cause may not be found despite extensive investigation in at least one fifth of children(4, 6, 7, 10).

Encephalitis is more prevalent among children than adults, with an estimated incidence of 4.0 – 12.6 per 100,000 person years for children in high income countries (7, 10-14). There is a substantially higher burden of childhood encephalitis in regions such as southeast Asia where the Japanese encephalitis virus is endemic(2, 8). Childhood encephalitis carries a significant mortality rate; this ranges from 5%-13%, dependent on setting and aetiology(4, 8, 10, 15, 16). Approximately half of children who survive an episode of encephalitis will have long term sequalae which may include neurological deficits, physical disability, cognitive impairment, neuropsychiatric disorders, and epilepsy(4, 8, 10, 15, 17-19). Childhood encephalitis is therefore associated with a high global economic, healthcare, and social burden(1, 3, 8, 15, 20).

Whilst there is good evidence for the efficacy of aciclovir in the management of encephalitis caused by herpes simplex virus (HSV) and varicella zoster virus (VZV)(21, 22), there are limited therapeutic options for other types of childhood encephalitis and the mainstay of treatment is supportive care. Treatment strategies for autoimmune encephalitis include methylprednisolone, plasma exchange and intravenous immunoglobulin, but the recommendations for their use are based largely on studies in individuals with specific types of autoimmune encephalitis, retrospective cohort studies and expert opinion(23-26). Furthermore, these therapies are often only implemented after a definitive autoimmune cause for encephalitis has been identified or all alternative diagnoses, including infectious, have been ruled out.

IVIG is used successfully in other inflammatory and neurological conditions in children(27, 28), however, there have been no high-quality studies to support or refute its use in children with all types of encephalitis (29, 30). Inflammation of the brain parenchyma is the common cause of altered neurological function in encephalitis, regardless of the aetiology, and it may therefore be postulated that interventions which attenuate the inflammation early in the illness are likely to have the greatest efficacy in reducing the severity of the acute illness, mortality and neurological sequalae of childhood encephalitis.

In this study, we set out to establish if early IVIG treatment, in addition to standard care, improves outcomes for children with encephalitis of all aetiologies.

Methods

Study Design

IgNiTE was a randomised, double blinded, parallel arm, placebo-controlled study to compare early IVIG treatment with placebo in the treatment of childhood encephalitis in individuals aged 6 months to 16 years. It was conducted across 21 National Health Service (NHS) hospitals in the UK. Participants were followed up for 12 months after randomisation, with outcomes assessed during the acute admission, at 4-8 weeks after discharge from acute care, at 6 months after randomisation, and 12 months after randomisation.

The trial was prospectively registered with ClinicalTrials.gov (identifier NCT 02308982) on 5 December 2014. The trial was assigned an International Standard Randomised Controlled Trial Number on 24 June 2015 (ISRCTN 15791925), and a European Clinical Trials Database number (2014-002997-35). A Trial Steering Committee (TSC) was established to oversee the trial, and an independent Data Monitoring and Ethics Committee (DMEC) was set up to monitor the safety, efficacy, and overall conduct of the study.

For peer review only - http://bmjopen.bmj.com/site/about/guidelines.xhtml

The original trial protocol was published on 03 November 2016(31). The protocol was amended after the early termination of the trial to remove endpoints which could not be derived from the data collected and to update the statistical analysis section; the amended protocol is available in the supplementary material.

Participants

Eligible participants were hospitalised children aged between 6 weeks and 16 years who met the case definition for encephalitis based on the consensus definition by the International Encephalitis Consortium(32), where written informed consent was obtained from parents or guardians, and assent was given if appropriate.

Exclusion criteria were a high clinical suspicion of bacterial meningitis; prior receipt of IVIG during the admission or known contraindication to IVIG; traumatic brain injury; history of metabolic encephalopathy; stroke, toxic or hypertensive encephalopathy; pre-existing demyelinating disorder; significant renal impairment; hypercoagulable state; hyperprolinaemia; participation in another research trial involving an immunomodulatory treatment; pregnancy; any significant disease or disorder which may put the participants at risk because of participation in the trial, influence the result of the trial, or the participant's ability to participate in the trial; involvement in another research trial involving an investigational medicinal product which has potential immunomodulatory or neuroprotective effects.

Intervention

Two doses of 1g/kg/dose of either IVIG or a matching volume of placebo were given 24-36 hours apart, with the first dose administered as soon as possible after enrolment and within five working days from the suspicion of an encephalitis diagnosis.

The active treatment (IVIG) used in the study was Privigen (100mg/ml solution), manufactured and provided by CSL Behring. The placebo was a mixture of 0.9% saline and 0.1% human albumin solution, manufactured at the Royal Broadgreen and Liverpool Aseptic Production Unit, Liverpool, UK under cGMP conditions and its Manufacturer's Importer's Authorisation (IMP) licence.

Randomisation and blinding

Participants were randomised 1:1 to IVIG or placebo treatment after consent was obtained. Randomisation was stratified by age group (< 1 year, 1-4 years, 5-9 years, 10-14 years and ≥15 years) and steroid treatment at the time of randomisation, using stratified block randomisation with randomly varying block sizes. Randomisation was performed using a secure web-based randomisation system (Sortition®) which was developed by the Clinical Trials Unit in the Nuffield Department of Primary Care Health Sciences, University of Oxford.

Participants, their parents or guardians, clinical staff, and all study staff (including staff involved in recruitment, administration of study treatment, data collection and entry, and laboratory analyses) were blind to the treatment allocation through the entire study period. Study monitors who were independent of the study and all site pharmacists were unblinded to ensure dispensing of the correct allocation and robust investigational medicinal product (IMP) management at each study site. The placebo and IVIG were visually identical, due to the additional of 0.1% human albumin solution to 0.9% in the placebo.

Primary Outcome

The primary outcome was good recovery, which was defined as a score of 2 or less on the paediatric version of the Glasgow Outcome Score Extended (GOS-E Peds) at 12 months after randomisation.

For peer review only - http://bmjopen.bmj.com/site/about/guidelines.xhtml

The GOS-E Peds is based on the GOS-E, a gold standard for measuring outcomes in adults with traumatic brain injury. It is a validated tool for use in children, and provides a developmentally appropriate structured interview necessary to evaluate children across different age groups(33). Participants were assigned a GOS-E Peds score: 1-Upper Good Recovery, 2-Lower Good Recovery, 3-Upper Moderate Disability, 4-Lower Moderate Disability, 5-Upper Severe Disability, 6-Lower Severe Disability, 7-Vegetative State, and 8-Death. 'Good recovery' was defined as a GOS-E Peds score of ≤ 2, and a score of >2 indicated 'poor recovery'.

Secondary outcomes

Secondary clinical outcomes included admission to intensive care unit, requirement for invasive ventilation, length of acute hospital stay, new diagnoses of epilepsy and need for anti-epileptic treatment in the 12 months after randomisation.

Secondary neurological and functional outcomes comprised GOS-E Peds assessment at 6 months after randomisation, and Liverpool Outcome Score (LOS) assessment, Pediatric Quality of Life Score (PedsQL) assessment, Gross Motor Function and Classification System (GMFCS) assessment, Strengths and Difficulty Questionnaire (SDQ) assessment and Adaptive Behavior Assessment System – second edition (ABAS-II) assessment at 4-8 weeks after discharge from acute care and at 12 months after randomisation.

Secondary neuropsychological outcomes were cognitive assessment at 12 months after randomisation using the age-appropriate scales: (i) Bayley Scales of Infant and Toddler Development, third edition (1 to 2 years 5 months); (ii) Wechsler Preschool Primary Scale of Intelligence IV (2 years 6 months to 5 years 11 months), and (iii) Wechsler Intelligence Scale for Children V (6 years to 16 years 11 months).

The secondary neuroimmunology outcome was identification of autoantibodies. The antibodies tested for were for antibodies against live neurons, Aquaporin 4 (AQP4), N-methyl-D-aspartate receptor, Myelin oligodendrocyte glycoprotein (MOG), leucine-rich, glioma inactivated 1 (LGI1), and Contactin-associated protein-like 2 (CASPR2).

Secondary neuroimaging outcomes comprised assessment of computerised tomography (CT) or magnetic resonance imaging (MRI) brain scans performed as part of routine care during the acute illness, and follow up scans performed at 6 months after randomisation in a subset of participants, where consent was provided.

Secondary safety outcomes included safety data obtained throughout the study, and a full blood count performed for all participants 24-48 hours following the second dose of the study treatment to monitor for haemolysis which has previously been described with high concentrations of IVIG treatment(34). Safety data comprised adverse events (AEs) and adverse events of special interest (AESIs) occurring in the first five days following receipt of each dose of the study drug, serious adverse events (SAEs) occurring up until 6 months after randomisation and serious adverse reactions (SARs) occurring throughout the study period. Information on any deaths occurring up to 12 months after randomisation was also collected.

Further information regarding to the secondary outcomes is provided in the Supplementary material.

Protocol Amendments

The IgNiTE study was halted in October 2017 after the withdrawal of funding due to slower than anticipated recruitment. This was despite the proposal of alternative strategies to deliver on the study objectives, including revision of the recruitment timeline to ensure that

For peer review only

For peer review only - http://bmjopen.bmj.com/site/about/guidelines.xhtml

the objectives of this important clinical study could be met. Where possible, follow up activities were completed for all participants who were already enrolled into the trial, as per the protocol. The protocol was amended to remove endpoints which could not be derived from the data collected and to update the statistical analysis section.

Statistical analysis

A sample size of 308 participants recruited over a 24 month period (154 per group, with approximate 10% attrition rate) was planned to achieve 90% power (at 5% level of significance) to detect at least a 20% clinically significant treatment difference from 43% in the 'good recovery' rate (defined as a GOS-E Peds score of ≤ 2) by 12 months after randomisation. This was based on the results of a large observational study on autoimmune encephalitis(26).

At the time the trial was halted, only 18 participants had been recruited. The trial was therefore underpowered to perform hypothesis testing of outcomes, subgroup comparisons or sensitivity analyses. Therefore, all analyses performed were descriptive. The analyses were performed on the intention-to-treat population;this included all 18 participants who were randomised. In the analysis of the adverse events, the population analysed were the 16 participants who received study treatment.

Patient and public involvement (PPI)

The Encephalitis Society was involved in the planning of this study, and the training of research nurses and study recruiters. A representative of The Encephalitis Society was on the Trial Management Group and provided a patient-centred research perspective to the study design and conduct. PPI groups were consulted in the development of the essential documents for the study including the participant information sheet and consent forms. Three PPI representatives with previous personal experiences of encephalitis sat on the Trial Steering Committee (TSC) and contributed to providing overall oversight of the study. Study update meetings were held to which patients previously affected by encephalitis were invited to share their experiences with the study team.

Results

Participants

A total of 884 patients were screened for eligibility between 23rd December 2015 and 26th September 2017 across 21 NHS hospitals, of whom 18 participants were enrolled and randomised across 12 hospital. 10 participants were assigned to IVIG treatment, and 8 patients were assigned to placebo. The study flow diagram is shown in Figure 1.

Table 1 summarises the baseline characteristics of participants by treatment arm. The mean age of the participants was 4.09 years (interquartile range (IQR) 2.0 – 11.8), 44% were male, and 89% were of White ethnicity.

Baseline characteristic	IVIG (n=10)	Placebo (n=8)	All (n=18)

For peer review only - http://bmjopen.bmj.com/site/about/guidelines.xhtml

**Age at randomisation (years)**	Median (IQR)	5.55 (1.52, 11.8)	4.09 (2.71, 9.64)	4.09 (2.0, 11.8)
**Sex**	Male	4 (40%)	4 (50%)	8 (44.4%)
	Female	6 (60%)	4 (50%)	10 (55.6%)
**Ethnicity**	White	8 (80%)	8 (100%)	16 (88.9%)
	Asian	1 (10%)	0 (0%)	1 (5.6%)
	Missing	1 (10%)	0 (0%)	1 (5.6%)
**History of immunocompromise**	No	9 (90%)	7 (87.5%)	16 (88.9%)
	Missing	1 (10%)	1 (12.5%)	2 (11.1%)
**Previous diagnosis of encephalitis**	No	9 (90%)	7 (87.5%)	16 (88.9%)
	Missing	1 (10%)	1 (12.5%)	2 (11.1%)
**History of encephalopathic illness**	No	9 (90%)	7 (87.5%)	16 (88.9%)
	Missing	1 (10%)	1 (12.5%)	2 (11.1%)
**Pre-existing diagnosis of epilepsy**	No	9 (90%)	7 (87.5%)	16 (88.9%)
	Missing	1 (10%)	1 (12.5%)	2 (11.1%)

Table 1: Baseline characteristics of enrolled participants

Primary Outcome

At 12 months after randomisation, nine participants (50%; IVIG n=5/10 [50%]; placebo n=4/8 [50%]) made a good recovery, defined as a GOS-E Peds score of $\leq$ 2. Five participants (28%; IVIG n=3/10 [30%], placebo n=2/8 [25%]) made a poor recovery, defined as a GOS-E Peds score of >2. Four participants (22%; IVIG n=2/10 [20%], placebo n=2/8 [25%]) did not undergo a GOS-E Peds assessment at this timepoint. Table 2 displays these results.

	**IVIG (N = 10)**	**Placebo (N = 8)**	**Overall N = 18)**
**GOSE-Peds Score**			
1.Upper good recovery	4 (40%)	4 (50%)	8 (44%)
2.Lower good recovery	1 (10%)	0 (0%)	1 (6%)
5.Upper severe disability	1 (10%)	1 (13%)	2 (11)
6.Lower severe disability	2 (20%)	1 (13%)	3 (17%)
Participants with missing data due to being withdrawn or lost to follow up	2 (20%)	2 (25%)	4 (22%)

Table 2: GOS-E Peds scores at 12 months after randomisation

Secondary Outcomes

For peer review only - http://bmjopen.bmj.com/site/about/guidelines.xhtml

Clinical Outcomes
Ten participants (56%; IVIG n=5/10 [50%], placebo=5/8 [63%]) were admitted to intensive care during their acute admission with encephalitis, as shown in Table 3. Nine of these participants (90%; IVIG n=4/5 [80%], placebo n=5/5 [100%]) required invasive ventilation, for a median duration of 2 days (IQR 2.0 - 3.0). The median length of stay on intensive care was 4.5 days (IQR 3.0 - 6.8) and the overall median length of hospitalisation for acute care was 11 days (IQR 7.8 - 19.5).

Three participants (17%; IVIG n=1/10 [10%], placebo n=2/8 [25%]) had a new diagnosis of epilepsy during the study period. Five participants (28%; IVIG n= 2/10 [20%], placebo n=3/8 [38%]) had incomplete data for this outcome.

Outcome		IVIG (N = 10)	Placebo (N = 8)	Overall (N = 18)
**During hospital stay**				
Duration of ventilation	Median (IQR)	2.5 (2.0, 3.5) [n = 4]	2.0 (2.0, 3.0) [n = 5]	2.0 (2.0, 3.0) [n = 9]
Length of ICU stay	Median (IQR)	4.0 (3.0, 6.0) [n = 5]	5.0 (2.0, 10.0) [n = 5]	4.5 (3.0, 6.8) [n = 10]
Length of hospitalisation for acute care	Median (IQR)	12.0 (8.0, 27.0) [n = 9]	8.0 (6.5, 14.0) [n = 7]	11.0 (7.8, 19.5) [n = 16]
**6 months post randomisation**				
New diagnosis of epilepsy since discharge	n (%)	1 (10%)	1 (13%)	2 (11%)
Anti-epileptic treatment since discharge	n (%)	1 (10%)	1 (13%)	2 (11%)
**12 months post randomisation**				
New diagnosis of epilepsy since discharge	n (%)	0 (0%)	1 (13%)	1 (6%)
Anti-epileptic treatment since discharge	n (%)	0 (0%)	0 (0%)	0 (0%)

Table 3: Secondary clinical outcomes

nly

GOS-E Peds assessment at 6 months
Fifteen participants underwent GOS-E Peds assessment at 6 months after randomisation. Eight participants (44%; IVIG n=4/10 [40%], placebo n= 4/8 [50%]) made a good recovery and seven participants (39%; IVIG n=4/10 [50%], placebo n=3/8 [38%]) made a poor recovery at this timepoint, as shown in Table 4.

Liverpool Outcome Score (LOS)
Fifteen participants had a LOS assessment at 4-8 weeks after discharge from acute care. Five participants (28%; IVIG n=3/10 [30%], placebo n=2/8 [25%]) made a full recovery, defined as a LOS of >4. Ten participants (56%; IVIG n= 5/10 [50%], placebo n=5/8 [63%]) had minor to severe sequelae. Table 4 displays the breakdown of these results.

Fourteen participants had a LOS assessment at 12 months after randomisation. Six participants (33%; IVIG n= 4/10 [40%], placebo n=2/8 [25%]) had made full recovery at this

For peer review only - http://bmjopen.bmj.com/site/about/guidelines.xhtml

timepoint, and eight participants (44%; IVIG n=4/10 [40%], placebo n=4/8 [50%]) had minor to severe sequelae.

Paediatric Quality of Life Assessment (PedsQL)
Seven participants (39%; IVIG n=5/10 [50%], placebo n=2/8 [25%]) had a PedsQL assessment at 4-8 weeks after discharge from acute care, and eight participants (44%; IVIG n=6/10 [60%], placebo n=2/8 [25%]) had a PedsQL assessment at 12 months after randomisation. At 4-8 weeks after discharge from acute care, the mean PedsQL score was 77.9 (standard deviation (SD) 11.10) and 56.5 (SD 7.8) for the IVIG and placebo group, respectively. At 12 months, mean PedsQL scores were 79.9 (SD 21.6) and 63.7 (SD 30.1) for the IVIG and placebo groups, respectively. This data is displayed in Table 4.

Gross Motor and Function Classification System (GMFCS)
Seven participants underwent a GMFCS assessment at 4-8 weeks after discharge from acute care, and eight participants underwent assessment at 12 months after randomisation. At 4-8 weeks after discharge, all seven participants assessed (39%; IVIG n=5/10 [50%]; placebo n=2/8 [25%]) had mild impairment of gross motor functioning. At 12 months after randomisation, all eight participants (44%; IVIG n=6/10 [60%]; placebo n=2/8 [25%]) experienced mild or severe impairment of gross motor function, as demonstrated by Table 4.

Strengths and difficulties assessment (SDQ)
SDQ results were available for seven participants (39%; IVIG n=5/10 [50%], placebo n=2/8 [25%]) at 4-8 weeks after discharge from acute care and eight participants (44%; IVIG n=6/10 [60%], placebo n=2/8 [25%]) at 12 months after randomisation.

At 4-8 week after discharge from acute care, five participants (28%; IVIG n=4 [40%]; placebo n=1 [13%]) had a close to average SDQ score, one participant (6%; IVIG n=1/10 [10%]) had a slightly raised SDQ score and one participant (6%; placebo n=1/8 [13%]) had a very high SDQ score. At 12 months after randomisation, the same number of participants had a close to average score and slightly raised score, but two participants (11%; IVIG n=1/10 [10%], placebo n=1/8 [13%]) had a very high SDQ score.

Adaptive Behaviour Assessment System – Second Edition (ABAS-II)
Eight participants had an ABAS-II assessment at 4-8 weeks after discharge from acute care, and seven participants had an ABAS-II assessment at 12 months after randomisation (see Table 4). At 4-8 weeks after discharge, five participants (28%; IVIG n=4/10 [40%], placebo n=1/8 [13%]) had an ABAS-II score that was either similar or higher than the average score of the normative population, and three participants (17%; IVIG n=2/10 [20%], placebo n=1/8 [13%]) had a score that was lower than the average score. At 12 months after randomisation, the same number of participants had a score that was below the average at 12 months after randomisation, but four participants (22%; IVIG n=3/10 [30%], placebo n=1/8 [13%]) had a score that was either similar or higher than the average score at this timepoint.

Outcome	4-8 weeks post discharge		12 months post randomisation

For peer review only - http://bmjopen.bmj.com/site/about/guidelines.xhtml

	IVIG (N=10)	Placebo (N=8)		IVIG (N=10)	Placebo (N=8)
**LOS**					
2.Severe sequelae	2 (20%)	2 (25%)		2 (20%)	2 (25%)
3.Moderate sequelae	2 (20%)	3 (38%)		1 (10%)	1 (13%)
4.Minor sequelae	1 (10%)	0 (0%)		1 (10%)	1 (3%)
5.Full recovery	3 (30%)	2 (25%)		4 (40%)	2 (25%)
Missing data due to withdrawal or loss to follow up of participant	1 (10%)	1 (13%)		2 (20%)	2 (25%)
Missing data – assessment not performed	1 (10%)	0 (0%)		0 (%)	0 (%)
**PedsQL**					
Mean (SD)	77.9 (11.1)	56.5 (7.8)		79.9 (21.6)	63.7 (30.1)
Missing data due to withdrawal or loss to follow up of participant	1 (10%)	1 (13%)		2 (20%)	2 (25%)
Missing data – assessment not performed	4 (40%)	5 (63%)		2 (20%)	4 (50%)
**SDQ**					
Close to average	4 (40%)	1 (13%)		4 (40%)	1 (13%)
Slightly raised	1 (10%)	0 (0%)		1 (10%)	0 (0%)
Very high	0 (0%)	1 (13%)		1 (10%)	1 (13%)
Missing data due to withdrawal or loss to follow up of participant	1 (10%)	1 (13%)		2 (20%)	2 (25%)
Missing data – assessment not performed	4 (40%)	5 (63%)		2 (20%)	4 (50%)
**ABAS**					
Very Superior	0 (0%)	0 (0%)		1 (10%)	0 (0%)
Superior	1 (10%)	0 (0%)		1 (10%)	0 (0%)
Above average	1 (10%)	0 (0%)		1 (10%)	0 (0%)
Average	2 (20%)	1 (13%)		0 (0%)	1 (13%)
Below average	0 (0%)	1 (13%)		1 (10%)	0 (0%)
Borderline	1 (10%)	0 (0%)		0 (0%)	0 (0%)
Extremely low	1 (10%)	0 (0%)		1 (10%)	1 (13%)
Missing data due to withdrawal or loss to follow up of participant	1 (10%)	1 (13%)		2 (20%)	2 (25%)
Missing data – assessment not performed	3 (30%)	5 (63%)		3 (30%)	4 (50%)
**GMFCS***					
Mild	5 (50%)	2 (25%)		6 (60%)	1 (13%)
Severe				0 (0%)	1 (13%)
Missing data due to withdrawal or loss to follow up of participant	1 (10%)	1 (13%)		2 (20%)	2 (25%)
Missing data – assessment not performed	4 (40%)	5 (63%)		2 (20%)	4 (50%)
**GOSE-Peds at 6 months post randomisation**					

	IVIG (N=10)	Placebo (N=8)
1.Upper good recovery	4 (40%)	4 (50%)
3.Upper moderate disability	1 (10%)	1 (13%)
5.Upper severe disability	0 (0%)	1 (13%)
6.Lower severe disability	3 (30%)	1 (13%)
Missing data due to withdrawal or loss to follow up of participant	2 (20%)	1 (13%)
Missing data – assessment not performed	0 (0%)	0 (0%)

For peer review only

For peer review only - http://bmjopen.bmj.com/site/about/guidelines.xhtml

Table 4: Secondary neurological and functional outcomes

Neuropsychology Outcomes
Thirteen participants (72%; IVIG n=8/10 [80%]; placebo n=5/8 [63%]) had a neuropsychology assessment at 12 months after randomisation by a blided assessor. Four of these participants (30%; IVIG n =2/8 [25%], placebo n=2/5 [40%]) were unable to complete the full battery of assessments due to attentional or behavioural needs.

Five participants (28%; IVIG n=4/10 [40%], placebo n=1/8 [13%]) had a score of ≥ 85 (indicating normal development) for full scale IQ (FSIQ), six (33%; IVIG n=4/10 [40%]; placebo n=2/8 [25%]) for verbal comprehension (VCI), five (28%; IVIG n=4/10 [40%], placebo n=1/8 [13%]) for visual spatial (VSI); four (22%; IVIG n=4/10 [40%]) for working memory (WMI); and four (22%; IVIG n=3/10 [30%]; placebo n=1/8 [13%]) for perceptual reasoning (PRI). Two participants (IVIG n=1, placebo n=1) were assessed using the Bayley scale of infant development, one participant (IVIG n=1) had severe neurodevelopmental impairment while the other (placebo n=1) had a normal neurodevelopmental outcome. These results are displayed in Table 5.

Participant	Bayley cognitive score	FSIQ	VCI	VSI/PRI	WMI	PSI
**Placebo arm**						
1	-	*	*	*	*	*
2	-	79	95	79	75	71
3	-	*	*	*	*	*
4	110	-	-	-	-	-
5	-	89	99	88	83	94
**IVIG arm**						
6	-	*	*	*	*	*
7	-	104	92	111	107	116
8	-	95	102	90	99	91
9	-	88	93	96	91	83
10	55	-	-	-	-	-
11	-	65	60	75	72	-
12	-	*	*	*	*	*
13	-	119	108	110	110	131

Key: Green = normal neurodevelopmental score, Yellow = mild impairment, Red = severe impairment. *Young person unable to complete full battery due to attention or behavioural needs

Table 5: Neuropsychology outcomes at 12 months after randomisation

Neuroimaging Outcomes
Nineteen acute neuroimaging scans were available for 13 participants (72%; IVIG n=8/10 [80%], placebo n=5/8 [63%]). Five of these scans (for five unique participants; IVIG n=2/8

For peer review only - http://bmjopen.bmj.com/site/about/guidelines.xhtml

[25%], placebo n=3/8 [38%]) had abnormal findings; all of these were MRI scans (see Supplementary Material Table 1). Four of the abnormal scans showed bilateral lesions.

There were nine follow up scans for eight unique participants (IVIG n=5/10 [50%], placebo n=3/8 [50%]); six of these scans (for five unique participants; IVIG n=3/5 [60%], placebo n=1/4 [25%]) were normal and unchanged from the acute scan. Three follow up scans (for three unique participants; IVIG n=2/5 [40%], placebo n=1/3 [33%]) had abnormal findings; two of these were unchanged from the acute scans and an acute scan was not available for comparison one participant.

Autoantibody Testing
Twelve participants (67%; IVIG n=7/10 [70%], placebo n=5/8 [63%]) had autoantibody testing. One participant (placebo n=1) was positive for LGI1 antibodies, and one participant (placebo n=1) was positive for MOG antibodies. Two additional participants (IVIG n=2) were positive for IgG binding to the surface of live neurons, indicating the presence of IgG antibodies binding to neurons, but negative for antibodies to the specific antigens tested, , indicating the presence of undefined IgG antibodies that could be pathogenic.

Safety Data
Ten serious adverse events occurred in three participants in the placebo group and none in the IVIG group. None of the SAEs were judged to be related to the study treatment. One participant in the IVIG group reported an adverse event of special interest; the participant developed a fever during the IVIG infusion, however, this was judged to be unrelated to the study treatment. None of the participants experienced haemolysis following receipt of two doses of study treatment. No deaths occurred during the study period.

Discussion

The IgNiTE study was terminated early due to slower than expected recruitment and was therefore unable to provide conclusive evidence regarding the efficacy of IVIG in the treatment of childhood encephalitis. Thus, it remains unknown whether early administration of IVIG in children with all-cause encephalitis offers clinical benefit.

Whilst the IgNITE study was unable to address the primary study objective, the results do provide evidence of the poor outcomes experiences by many children with encephalitis. Almost a third of participants made a poor recovery based on GOS-E Peds assessment at 12 months after randomisation. Other measures of neurological outcomes consistently demonstrated a heavy burden of disability; 44% of patients had minor to severe sequalae at 12 months according to the LOS assessment, and the same proportion of patients experienced mild or severe impairment of gross motor function at the same timepoint. The proportion of children with functional impairments on the SDQ and ABAS-II assessments at 12 months after randomisation was lower, but this was likely due to fewer participants completing these assessments.

The results also demonstrate the impact of childhood encephalitis on healthcare systems. Over half of participants required admission to intensive care during the acute illness, and 90% of these children were intubated. The overall median length of acute hospital care for participants was 11 days, compared with a mean length of hospital stay of 1.64 days for children and young people following an emergency admission in the UK(35). Furthermore, given the high proportion of participants with lasting disability, many children with encephalitis are likely require ongoing non-acute hospital care for neurorehabilitation.

These data are consistent with previous studies of childhood encephalitis in high-income settings. In a prospective Australian study involving 287 children with encephalitis, 49% of

For peer review only - http://bmjopen.bmj.com/site/about/guidelines.xhtml

children required admission to intensive care, median length of hospitalisation was 11 days and 27% of children had moderate to severe neurodisability at hospital discharge(4). Of note, they used the adult Glasgow Outcome Score (GOS) tool for assessment of outcomes and did not capture children with mild to moderate neurodisability, which may explain the lower proportion of children with reported neurodisability compared with the IgNiTE study. A meta-analysis evaluating long-term outcomes of childhood encephalitis reported 47% of children to have long-term sequalae in studies in high income countries, although there was no standardised definition of sequalae used across these studies(17).

Limitations of the study

The main limitation of the IgNiTE study is that the predefined sample size was not met, and the primary study objective was therefore not achieved. The study initially planned to recruit 308 participants over a 24 month period. The sample size calculation was based on the anticipated number of annual encephalitis hospital admissions in the UK and the anticipated treatment effect of IVIG, based on a large observational study on autoimmune encephalitis (36) (26). However, recruitment to the study was slower than expected. Of the 884 children assessed for eligibility, 63% (561) were excluded because they did not meet the case definition for encephalitis, suggesting that the use of strict diagnostic criteria may have precluded the inclusion of some children with clinically suspected encephalitis. A further 12.5% were excluded due to insufficient clinical results being available to satisfy the eligibility criteria within the time frame for participant enrolment. The initial screening form used did not capture the reason for exclusion, hence this was not recorded for the first 10% of children assessed for eligibility.

Overall, 13% (115) of children were assessed to meet to inclusion criteria, but 55% (63) of these children fulfilled exclusion criteria and were thus ineligible. The main reasons for exclusion were prior or planned IVIG treatment as part of routine care (32%), and study timeline restrictions (24%). The use of IVIG as part of routine care demonstrates that some clinicians were already convinced of the benefit of IVIG in childhood encephalitis despite the lack of high-quality evidence and the fact that at the time the trial was undertaken, IVIG was not commissioned for routine use in acute childhood encephalitis. This highlights the importance of ensuring that there is equipoise amongst treating clinicians when conducting randomised controlled trials.

Recruitment to the trial was also impacted by a lower than anticipated consent rate. Of the 52 children who were eligible for enrolment, participation was declined in 65% of cases. This is not unexpected given the requirement for parents or guardians to provide informed consent at an exquisitely sensitive time for the family. Other factors which may have contributed to the low consent rate include the limited time frame for enrolment and the trial duration(37).

Finally, recruitment was impacted by delays in the participating NHS hospitals opening as recruitment sites, due primarily to shortages of research personnel and delays in local approval processes. Nine of the 21 participating hospitals did not recruit any particitpants during the study; five of these hospitals were open to recruitment for six months or less.

Lessons learned and future research

Further research is required to establish whether early IVIG is of therapeutic benefit in the treatment of childhood encephalitis, irrespective of the underlying aetiology. The IgNiTE study demonstrated the feasibility of conducting a randomised controlled trial to investigate this important question. Future studies should anticipate the recruitment challenges

For peer review only - http://bmjopen.bmj.com/site/about/guidelines.xhtml

discussed above and consider strategies such as incorporating a pilot phase, using less strict entry criteria, allowing a wider time frame in which participants can be enrolled, and adopting approaches to optimise consent rates in eligible patients.

Conclusion

The IgNiTE study was terminated prematurely due to slow recruitment and therefore did not reach the pre-determined sample size required to evaluate the effect of IVIG compared to placebo in childhood encephalitis.. However, the study results support existing evidence of poor neurological outcomes in many children with encephalitis. This provides further compelling evidence of the need for better treatments in childhood encephalitis. Future studies are required to establish if treatment with IVIG is of benefit in children with encephalitis of all causes. Such studies should take into account the challenges encountered and lessons learnt from the IgNiTE study.

What is already known on this topic

- Childhood encephalitis is associated with poor outcomes for many children, despite current treatment strategies
- The benefit of IVIG in children with all types of encephalitis is unclear

What this study adds

- The study provides supportive evidence of the poor long term neurological and functional outcomes for many children with encephalitis
- The study was unable to answer the question of whether IVIG improves outcomes for children with encephalitis, but there are important lessons to learnt for future studies investigating this question

Ethics Statements

The study was approved by the UK National Research Ethics Service (NRES) committee (South Central—Oxford A; REC 14/SC/1416). Written approval from local Research and Development (R&D) departments at each participating site was obtained before recruitment was commenced at each site.
This study was conducted in accordance with the Declaration of Helsinki (1996), in full conformity with the International Conference on Harmonisation of Technical Requirements for Registration of Pharmaceuticals for Human Use (ICH) Guidelines for Good Clinical Practice (CPMP/ICH/135/95 July 1996), the Research Governance Framework, and the Medicines for Human Use (Clinical Trial) Regulations 2004.

Funding statement

This project was funded by the National Institute for Health Research (NIHR) Efficacy and Mechanism Evaluation programme (grant number 12/212/15). The investigational medicinal product (IMP), intravenous immunoglobulin (Privigen) was provided by CSL Behring.

Data sharing statement

All data requests should be submitted to the corresponding author for consideration. Access to an anonymised dataset may be granted following review.

Acknowledgements

The authors would like to thank all the participants, families, and health-care professionals for their involvement in the study.

For peer review only - http://bmjopen.bmj.com/site/about/guidelines.xhtml

Author Statement

Authors's individual contributions

The trial was conceptualised by MP and AJP, and designed with input from ML, MA, MS, MAI, L-MY, RK and TS. MP, MS, RK, TS, WKC, CAC, ML, MA, AV, VG, AE, L-MY and AJP are named investigators on the IgNiTE trial. Each co-author provided specific additional contributions within their area of expertise; paediatric neurology (MP, RK, ML, MA), paediatric infectious diseases (MS, AJP), paediatric neuropsychology (VG), neuroimaging (CAC, WKC), neuroimmunology (AV), statistics (L-MY, LC, XL) and patient group (AE). MAI is the lead doctor for the trial and LW is the lead nurse for the trial. MH prepared the first version of the manuscript. All authors contributed to the manuscript and have approved the final manuscript for publication.

The IgNiTE Study Team

Trial Management Group

Prof Sir Andrew J Pollard (Chief Investigator), Dr Ming Lim, Prof Tom Solomon, Dr Rachel Kneen, Dr Michael Absoud, Dr Mike Pike, Dr Manish Sadarangani, Dr Kling Chong, Dr Chris A. Clark, Dr Victoria Gray

Trial coordinating team (Oxford)

Dr Mildred A Iro (Clinical ResearchFfellow), Louise Willis (Research Nurse/Project Manager), David Kerr, Sophie Bradshaw, Svetlana Milca, Simon Kerridge, Emma Plested, Yama Mujadidi

IMP management

Shakeel Herwitker, Mandy Wan

Trial Steering Committee

Dr Claire Cameron, Dr Ming Lim, Dr Adilia Warris, Dr Federico Martinon-Torres, Mike Bale, Sonia Bale, Alan Percival, and Dr Ava Easton

Data Monitoring and Ethics Committee

Dr Charles Warlow (Chairperson), Dr Jo Haviland (statistician), Dr David Pace and Dr Simon Nadel

Statistics team

Dr Ly-Mee Yu, Dr Meryn Voysey, Dr Xinxue Liu, Liberty Cantrell

Laboratory team (Oxford)

Sagida Bibi, Amy Beveridge, Amber Thompson, Daniel O'Connor, Prof Sarosh Irani, Dr Patrick Waters

Neuropsychological assessment support

Lauren Burke, Victoria Gray

Oxford Vaccine Group

Emma Plested, Parvinder Alley

Recruitment sites

Name of hospital	Principal Investigator

For peer review only

Oxford University Hospitals	*Andrew Pollard*
Great Ormond Street Hospital	*Sanjay Bhate*
Alder Hey Children's Hospital	*Rachel Kneen*
St George's Hospital	*Paul Heath*
Barts and the London (Royal London)	*Anna Riddell*
Guy's and St Thomas's - Evelina	*Ming Lim*
Sheffield Children's Hospital	*Archana Desurkar*
NHS Grampian	*Elma Stephen*
Heart of England	*Steve Welch*
Pennine - North Manchester Children's Hospital	*Paddy McMaster*
Ninewells (Tayside Health Board)	*Alice Jollands*
Royal Hospital for Sick Children, Edinburgh	*Jay Shetty*
University Hospitals Bristol	*Kayal Vijayakumar*
Imperial – St Mary's Hospital	*Leena Mewasingh*
Nottingham University Hospitals	*William Whitehouse*
Hull Royal Infirmary	*Vishal Mehta*
University Hospitals of North Midland	*John Alexander*
Leeds Teaching Hospital	*John Livingston*
Royal Preston Hospital	*Christian de Goede*
York Teaching Hospital	*Dominic Smith*
Royal Cornwall Hospitals	*Andrew Collinson*

Declared competing interests of authors

Mildred A Iro was a trainee member on the NIHR Efficacy and Mechanism Evaluation Programme Funding Committee from October 2020 to October 2021. Michael Absoud has received a grant from the NIHR in the last 36 months, for research unrelated to the submitted work. MS has been an investigator on projects funded by GlaxoSmithKline, Merck, Moderna, Pfizer, Sanofi-Pasteur, Seqirus, Symvivo and VBI Vaccines; all funds have been paid to his institute. Ava Easton is Chief Executive of the Encephalitis Society, which has previously received grants from CSL Behring (UK). Ming Lim has received grants from the GOSH charity, Boston Children's Hospital Research Fund and Action Medical Research in the last 36 months, all for research unrelated to the submitted work. Ming Lim is co-chair of the European Paediatric Neurology Education and Training Board and works for an institution which holds research accounts with Roche (Switzerland), Octapharma (Switzerland) and Novartis (Switzerland). Tom Solomon is supported by the NIHR Health Protection Research Unit in Emerging and Zoonotic Infections, NIHR Programme Grant for Applied Research, NIHR Global Health Research on Brain Infections and the European Union's Horizon 2020 research and innovation program ZikaPLAN. Tom Solomon is a consultant for the MHRA Vaccine Benefit Risk Expert Working Group. Angela Vincent is a consultant for Aspen NewCo Inc and has received honoraria from UCB and Alexion. Ly-Mee Yu was a member of the NIHR Health Technology Assessment Efficient Study Designs from November 2015 to July 2016. Andrew J Pollard is chair of the Department of Health and Social Care's Joint committee on Vaccines and Immunisation (JCVI) and was a member of WHO's SAGE until 1st January 2022. Oxford University has entered a partnership with AstraZenenca on COVID-19 vaccines, but Andrew Pollard does not participate in the JCVI COVID-19 committee.

References

1. Tyler KL. Acute Viral Encephalitis. N Engl J Med. 2018;379(6):557-66.

For peer review only - http://bmjopen.bmj.com/site/about/guidelines.xhtml

2. Wang H, Zhao S, Wang S, Zheng Y, Wang S, Chen H, et al. Global magnitude of encephalitis burden and its evolving pattern over the past 30 years. J Infect. 2022;84(6):777-87.
3. Dr Julia Granerod AE, Dr Nicholas Davies, Dr Benedict Michael, Professor Tom Solomon, Dr Ava Easton. Encephalitis: an in-depth review and gap analysis of key variables affecting global disease burden. Encephalitis Society; 2022 July 21 2022.
4. Britton PN, Dale RC, Blyth CC, Clark JE, Crawford N, Marshall H, et al. Causes and Clinical Features of Childhood Encephalitis: A Multicenter, Prospective Cohort Study. Clin Infect Dis. 2020;70(12):2517-26.
5. Timothy A. Erickson EM, Flor M. Munoz, Timothy Lotze, Rodrigo Hasbun, Eric Brown,Kristy O. Murray, . Infectious and Autoimmune Causes of Encephalitis in Children. Pediatrics. 2020;145 (6).
6. de Blauw D, Bruning AHL, Busch CBE, Kolodziej LM, Jansen NJG, van Woensel JBM, et al. Epidemiology and Etiology of Severe Childhood Encephalitis in The Netherlands. Pediatr Infect Dis J. 2020;39(4):267-72.
7. George BP, Schneider EB, Venkatesan A. Encephalitis hospitalization rates and inpatient mortality in the United States, 2000-2010. PloS one. 2014;9(9):e104169.
8. Pommier JD, Gorman C, Crabol Y, Bleakley K, Sothy H, Santy K, et al. Childhood encephalitis in the Greater Mekong region (the SouthEast Asia Encephalitis Project): a multicentre prospective study. Lancet Glob Health. 2022;10(7):e989-e1002.
9. Granerod J, Crowcroft NS. The epidemiology of acute encephalitis. Neuropsychol Rehabil. 2007;17(4-5):406-28.
10. Wickström R, Fowler Å, Bogdanovic G, Bennet R, Eriksson M. Review of the aetiology, diagnostics and outcomes of childhood encephalitis from 1970 to 2009. Acta Paediatr. 2017;106(3):463-9.
11. Britton PN, Khoury L, Booy R, Wood N, Jones CA. Encephalitis in Australian children: contemporary trends in hospitalisation. Arch Dis Child. 2016;101(1):51-6.
12. Jmor F, Emsley HC, Fischer M, Solomon T, Lewthwaite P. The incidence of acute encephalitis syndrome in Western industrialised and tropical countries. Virol J. 2008;5:134.
13. Iro MA, Sadarangani M, Goldacre R, Nickless A, Pollard AJ, Goldacre MJ. 30-year trends in admission rates for encephalitis in children in England and effect of improved diagnostics and measles-mumps-rubella vaccination: a population-based observational study. Lancet Infect Dis. 2017;17(4):422-30.
14. Rantakallio P, Leskinen M, von Wendt L. Incidence and prognosis of central nervous system infections in a birth cohort of 12,000 children. Scand J Infect Dis. 1986;18(4):287-94.
15. Griffiths MJ, Lemon JV, Rayamajhi A, Poudel P, Shrestha P, Srivastav V, et al. The functional, social and economic impact of acute encephalitis syndrome in Nepal--a longitudinal follow-up study. PLoS Negl Trop Dis. 2013;7(9):e2383.
16. Granerod J, Ambrose HE, Davies NW, Clewley JP, Walsh AL, Morgan D, et al. Causes of encephalitis and differences in their clinical presentations in England: a multicentre, population-based prospective study. Lancet Infect Dis. 2010;10(12):835-44.
17. Khandaker G, Jung J, Britton PN, King C, Yin JK, Jones CA. Long-term outcomes of infective encephalitis in children: a systematic review and meta-analysis. Dev Med Child Neurol. 2016;58(11):1108-15.
18. Fowler A, Stödberg T, Eriksson M, Wickström R. Childhood encephalitis in Sweden: etiology, clinical presentation and outcome. Eur J Paediatr Neurol. 2008;12(6):484-90.

For peer review only - http://bmjopen.bmj.com/site/about/guidelines.xhtml

19. Fowler Å, Stödberg T, Eriksson M, Wickström R. Long-term Outcomes of Acute Encephalitis in Childhood. Pediatrics. 2010;126(4):e828-e35.
20. Deng X, Yan R, Li ZQ, Tang XW, Zhou Y, He H. Economic and disease burden of Japanese encephalitis in Zhejiang Province, 2013-2018. PLoS Negl Trop Dis. 2021;15(6):e0009505.
21. Sköldenberg B, Alestig K, Burman L, Forkman A, Lövgren K, Norrby R, et al. ACYCLOVIR VERSUS VIDARABINE IN HERPES SIMPLEX ENCEPHALITIS: Randomised Multicentre Study in Consecutive Swedish Patients. The Lancet. 1984;324(8405):707-11.
22. Whitley RJ, Alford CA, Hirsch MS, Schooley RT, Luby JP, Aoki FY, et al. Vidarabine versus acyclovir therapy in herpes simplex encephalitis. N Engl J Med. 1986;314(3):144-9.
23. Zuliani L, Nosadini M, Gastaldi M, Spatola M, Iorio R, Zoccarato M, et al. Management of antibody-mediated autoimmune encephalitis in adults and children: literature review and consensus-based practical recommendations. Neurol Sci. 2019;40(10):2017-30.
24. Dubey D, Britton J, McKeon A, Gadoth A, Zekeridou A, Lopez Chiriboga SA, et al. Randomized Placebo-Controlled Trial of Intravenous Immunoglobulin in Autoimmune LGI1/CASPR2 Epilepsy. Ann Neurol. 2020;87(2):313-23.
25. Nosadini M, Eyre M, Molteni E, Thomas T, Irani SR, Dalmau J, et al. Use and Safety of Immunotherapeutic Management of N-Methyl-d-Aspartate Receptor Antibody Encephalitis: A Meta-analysis. JAMA Neurol. 2021;78(11):1333-44.
26. Titulaer MJ, McCracken L, Gabilondo I, Armangué T, Glaser C, Iizuka T, et al. Treatment and prognostic factors for long-term outcome in patients with anti-NMDA receptor encephalitis: an observational cohort study. Lancet Neurol. 2013;12(2):157-65.
27. Oates-Whitehead RM, Baumer JH, Haines L, Love S, Maconochie IK, Gupta A, et al. Intravenous immunoglobulin for the treatment of Kawasaki disease in children. Cochrane Database Syst Rev. 2003;2003(4):CD004000.
28. Hughes RA, Swan AV, van Doorn PA. Intravenous immunoglobulin for Guillain-Barre syndrome. Cochrane Database Syst Rev. 2014;2014(9):CD002063.
29. Iro MA, Martin NG, Absoud M, Pollard AJ. Intravenous immunoglobulin for the treatment of childhood encephalitis. Cochrane Database Syst Rev. 2017;10:CD011367.
30. Wagner JN, Leibetseder A, Troescher A, Panholzer J, von Oertzen TJ. Efficacy and safety of intravenous immunoglobulins for the treatment of viral encephalitis: a systematic literature review. J Neurol. 2022;269(2):712-24.
31. Iro MA, Sadarangani M, Absoud M, Chong WK, Clark CA, Easton A, et al. ImmunoglobuliN in the Treatment of Encephalitis (IgNiTE): protocol for a multicentre randomised controlled trial. BMJ Open. 2016;6(11):e012356.
32. Venkatesan A, Tunkel AR, Bloch KC, Lauring AS, Sejvar J, Bitnun A, et al. Case definitions, diagnostic algorithms, and priorities in encephalitis: consensus statement of the international encephalitis consortium. Clin Infect Dis. 2013;57(8):1114-28.
33. Beers SR, Wisniewski SR, Garcia-Filion P, Tian Y, Hahner T, Berger RP, et al. Validity of a pediatric version of the Glasgow Outcome Scale-Extended. J Neurotrauma. 2012;29(6):1126-39.
34. Kahwaji J, Barker E, Pepkowitz S, Klapper E, Villicana R, Peng A, et al. Acute hemolysis after high-dose intravenous immunoglobulin therapy in highly HLA sensitized patients. Clin J Am Soc Nephrol. 2009;4(12):1993-7.
35. Kossarova EsKaL. Focus on: Emergency hospital care for children and young people. QualityWatch; 2017 April 2017.

For peer review only

36. Kneen R, Michael BD, Menson E, Mehta B, Easton A, Hemingway C, et al. Management of suspected viral encephalitis in children - Association of British Neurologists and British Paediatric Allergy, Immunology and Infection Group national guidelines. J Infect. 2012;64(5):449-77.
37. V Shilling PW, H Hickey, E Sowden,, RL Smyth BY. Processes in recruitment to randomised controlled trials of medicines for children (RECRUIT): a qualitative study. Health Technology Assessment. 2011;15.

Figure 1: Study flow diagram

For peer review only

Figures

Figure 1: Study flow diagram

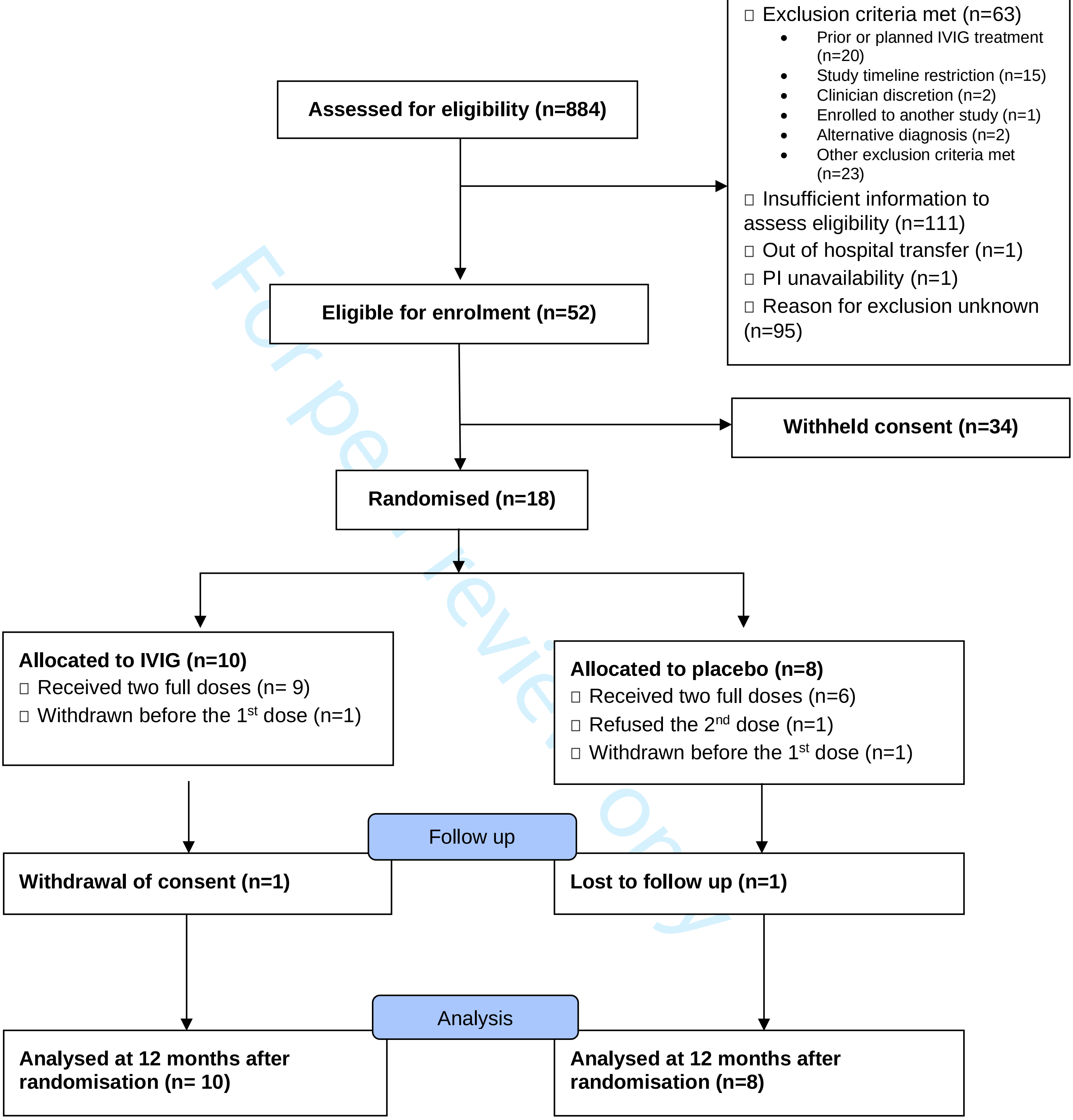

For peer review only

Supplementary Material

Secondary outcomes

Clinical

Secondary clinical outcomes were obtained from routinely collected medical information. These comprised admission to intensive care unit, invasive ventilation requirement, and length of hospital stay, defined as the number of days from admission to a recruiting hospital to discharge from acute care (i.e. not including days in hospital for neurorehabilitation). At 6 and 12 months after randomisation, information on new diagnosis of epilepsy and need for anti-epileptic treatment since discharge were collected.

Neurological and Functional

Secondary neurological outcomes were assessed using age appropriate questionnaires and outcome scores which comprised the GOS-E Peds (assessed at 6 months after randomisation), Liverpool Outcome Score (LOS), Pediatric Quality of Life Score (PedsQL), Gross Motor Function and Classification System (GMFCS), Strengths and Difficulty Questionnaire (SDQ), and Adaptive Behaviour Assessment System, second edition (ABAS-II), all assessed at 4-8 weeks after discharge from acute care and 12 months after randomisation.

The LOS is a validated tool for assessing level of disability after encephalitis in infants and children. It was originally designed to assess disease burden following JE and its use has been extended to other forms of encephalitis. For each participant, a total score (sum of scores for all questions) and an outcome score (the lowest score for any single question) were documented. Based on the outcome score only, participants were assigned to one of 5 outcome categories: 5-Full recovery, 4-Minor sequelae, 3-Moderate sequelae, 2-Severe sequelae, and 1-Death. 'Good recovery' was defined as a LOS of 5 and a score of ≤ 4 indicated 'poor recovery'.

The PedsQL is a brief measure of health-related quality of life comprised of 23 items assessing quality of life in 4 domains: physical functioning (8 items), emotional functioning (5 items), social functioning (5 items) and school functioning (5 items). Based on the scores in each domain, two summary scores (physical health and

For peer review only

psychosocial health summary scores) as well as a total scale score were computed. Total scale scores are presented. A higher total scale score indicates better quality of life.

The GMFCS is an assessment tool based on self-initiated movement and assesses motor function in three areas - walking, sitting, and standing. It uses 5 levels to describe the motor function limitations, taking into consideration age, the use of mobility aids and the quality of movement and is rated from Level 1 (walks without limitations) to Level 5 (transported in a manual wheelchair). Levels 1 and 2 have almost independent mobility while level 3 can move with assistive devices and levels 4 and 5 are significantly limited and dependent on their helpers for minor movements. A higher score describes worse dysfunction and less dependence during mobility as the level goes up. Gross motor function was categorised as mild (Levels 1 and 2), moderate (level 3) and severe (Levels 4 and 5).

The SDQ is a 25-item questionnaire comprising 5 scales of 5 items each focusing on difficulties relating to emotional functioning, conduct, hyperactivity, and interaction with peers. Scale scores and a total difficulties score (generated by summing the scores from all the scales except the prosocial scale) were documented. Based on the total difficulties score, SDQ scores were categorised into 4 bands: close to average, slightly lower, low, and very low, based on a UK community sample. For 2–4-year-old children, the close to average category contains 80%, the slightly raised category contains 12%, the high category contains 4%, and the very high category contains 4% of the population. For 4-17-year-old parent completed questionnaires, the close to average category contains 80%, the slightly raised category contains 10%, the high category contains 5%, and the very high category contains 5% of the sampled UK population.

The ABAS-II is an instrument used to evaluate adaptive skills that are important to everyday living and assesses three main domains: (i) Conceptual (summarises performance in the following skill areas - communication, functional academics, and self-direction), (ii) Social (leisure and social), and (iii) Practical (community use, home living, health and safety, self-care). The individual response provided for each skill area question was assigned a score. The total score allocated to each domain

For peer review only

was obtained by summing up the skills scores in that domain. Raw scores were converted into composite scores, with a population mean of 100 and a standard deviation of 15, with a lower score signifies worse adaptive behaviour. Composite scores were divided into the following categories based on percentiles (%) of the normative population: very superior > 130 (≥ 98%); superior 120–129 (91–97%); above average 110–119 (75–90%); average 90–109 (25–74%); below average 80–89 (9–24%); borderline 71–79 (3–8%); extremely low 70 or less (≤2%).

Neuropsychological

A blinded neuropsychology assessment was performed at 12 months after randomisation during which cognitive function was assessed using the following age appropriate scales: (i) Bayley Scales of Infant and Toddler Development, third edition (1 to 2 years 5 months); (ii) Wechsler Preschool Primary Scale of Intelligence IV (2 years 6 months to 5 years 11 months), and (iii) Wechsler Intelligence Scale for Children V (6 years to 16 years 11 months).

The Bayley Scales of Infant and Toddler Development (BSID-III) is a widely used and validated measure of cognitive functioning which produces three composite scores: cognitive scale, language scale (receptive and expressive), and motor scale (fine and gross). The Wechsler Preschool Primary Scale of Intelligence IV produces scores for: Verbal Comprehension (VCI), Visual Spatial (VSI), Fluid Reasoning, Working Memory (WMI), Processing Speed (PSI), and Full-scale IQ (FSIQ). The Wechsler Intelligence Scale for Children IV assesses general thinking and reasoning skills and is made up of 10 subtests, yielding 4 composite scores (Verbal Comprehension, Perceptual Reasoning (PRI), Working Memory, and Processing Speed). The Full-Scale IQ (composite score) is an average of these four scales.

Composite standard scores have a mean of 100 and SD of 15. Neurodevelopmental outcome was classified as (i) severe impairment (composite score of <70, >2SD below the mean), (ii) mild impairment (score of 70-84, >1SD below the mean) and (iii) normal neurodevelopmental (score of ≥ 85)

Neuroimmunology

For peer review only

Auto-antibody testing was performed by the clinical neuroimmunology service at the Nuffield Department of Clinical Neurosciences, Oxford.

Neuroimaging

Neuroimaging findings were obtained from clinical CT or MRI scans. In addition, an optional follow up research MRI scan was performed in a subset of participants, where consent was provided. Anonymised scans were analysed for the following:

Initial clinical scan(s):

- Proportion of participants with an abnormal scan
- Distribution of disease – structural and functional anatomy of lesion
- Subset of radiological features (mass effect, hydrocephalus, enhancement, other)

Follow up scan(s)

- Proportion of participants with an abnormal scan
- Lesion resolution/persisting disease
- Presence of new lesions
- Distribution of disease –structural and functional anatomy of lesion
- Subset of radiological features (mass effect, hydrocephalus, enhancement, other).

Supplementary Material Table 1: Baseline neuroimaging results summarising overall findings of acute scans

Participant Number	Type of scan	Overall assessment	Laterality of abnormality
1	MRI	Abnormal	Bilateral
2	MRI	Normal	N/A
3	MRI	Abnormal	Unilateral (Right)
4	CT scan	Normal	N/A
4	MRI	Abnormal	Bilateral
5	MRI	Normal	N/A
6	CT scan	Normal	N/A
6	MRI	Normal	N/A
7	MRI	Not available	Not available

For peer review only

8	MRI	Normal	N/A
8	MRI	Normal	N/A
9	MRI	Abnormal	Bilateral
10	CT scan	Normal	N/A
10	MRI	Abnormal	Bilateral
11	MRI	Normal	N/A
12	CT scan	Normal	N/A
12	CT scan	Normal	N/A
12	MRI	Normal	N/A
13	CT scan	Normal	N/A

Key: N/A= Not applicable

1. Kahwaji J, Barker E, Pepkowitz S, Klapper E, Villicana R, Peng A, et al. Acute hemolysis after high-dose intravenous immunoglobulin therapy in highly HLA sensitized patients. Clin J Am Soc Nephrol. 2009;4(12):1993-7.

For peer review only

CONSORT 2010 checklist of information to include when reporting a randomised trial*

Section/Topic	Item No	Checklist item	Reported on page No
**Title and abstract**			
	1a	Identification as a randomised trial in the title	1
	1b	Structured summary of trial design, methods, results, and conclusions (for specific guidance see CONSORT for abstracts)	2-3
**Introduction**			
Background and objectives	2a	Scientific background and explanation of rationale	3-4
	2b	Specific objectives or hypotheses	4
**Methods**			
Trial design	3a	Description of trial design (such as parallel, factorial) including allocation ratio	4
	3b	Important changes to methods after trial commencement (such as eligibility criteria), with reasons	6
Participants	4a	Eligibility criteria for participants	4
	4b	Settings and locations where the data were collected	4
Interventions	5	The interventions for each group with sufficient details to allow replication, including how and when they were actually administered	5
Outcomes	6a	Completely defined pre-specified primary and secondary outcome measures, including how and when they were assessed	5,6
	6b	Any changes to trial outcomes after the trial commenced, with reasons	6
Sample size	7a	How sample size was determined	6
	7b	When applicable, explanation of any interim analyses and stopping guidelines	-
Randomisation:			
Sequence generation	8a	Method used to generate the random allocation sequence	5
	8b	Type of randomisation; details of any restriction (such as blocking and block size)	5
Allocation concealment mechanism	9	Mechanism used to implement the random allocation sequence (such as sequentially numbered containers), describing any steps taken to conceal the sequence until interventions were assigned	5
Implementation	10	Who generated the random allocation sequence, who enrolled participants, and who assigned participants to interventions	5

For peer review only - http://bmjopen.bmj.com/site/about/guidelines.xhtml

Blinding	11a	If done, who was blinded after assignment to interventions (for example, participants, care providers, those assessing outcomes) and how	5
	11b	If relevant, description of the similarity of interventions	5
Statistical methods	12a	Statistical methods used to compare groups for primary and secondary outcomes	6
	12b	Methods for additional analyses, such as subgroup analyses and adjusted analyses	6
**Results**			
Participant flow (a diagram is strongly recommended)	13a	For each group, the numbers of participants who were randomly assigned, received intended treatment, and were analysed for the primary outcome	7
	13b	For each group, losses and exclusions after randomisation, together with reasons	7
Recruitment	14a	Dates defining the periods of recruitment and follow-up	7
	14b	Why the trial ended or was stopped	9
Baseline data	15	A table showing baseline demographic and clinical characteristics for each group	Table 1
Numbers analysed	16	For each group, number of participants (denominator) included in each analysis and whether the analysis was by original assigned groups	Figure 1
Outcomes and estimation	17a	For each primary and secondary outcome, results for each group, and the estimated effect size and its precision (such as 95% confidence interval)	Tables 2-5. Pages 7-9
	17b	For binary outcomes, presentation of both absolute and relative effect sizes is recommended	N/A
Ancillary analyses	18	Results of any other analyses performed, including subgroup analyses and adjusted analyses, distinguishing pre-specified from exploratory	N/A
Harms	19	All important harms or unintended effects in each group (for specific guidance see CONSORT for harms)	9
**Discussion**			
Limitations	20	Trial limitations, addressing sources of potential bias, imprecision, and, if relevant, multiplicity of analyses	10
Generalisability	21	Generalisability (external validity, applicability) of the trial findings	10,11
Interpretation	22	Interpretation consistent with results, balancing benefits and harms, and considering other relevant evidence	11
**Other information**			
Registration	23	Registration number and name of trial registry	1
Protocol	24	Where the full trial protocol can be accessed, if available	Supplementary material
Funding	25	Sources of funding and other support (such as supply of drugs), role of funders	11

For peer review only

*We strongly recommend reading this statement in conjunction with the CONSORT 2010 Explanation and Elaboration for important clarifications on all the items. If relevant, we also recommend reading CONSORT extensions for cluster randomised trials, non-inferiority and equivalence trials, non-pharmacological treatments, herbal interventions, and pragmatic trials. Additional extensions are forthcoming: for those and for up to date references relevant to this checklist, see www.consort-statement.org.

For peer review only
